# Exosome-mediated genetic reprogramming of tumor-associated macrophages by exoASO-STAT6 leads to potent monotherapy antitumor activity

**DOI:** 10.1126/sciadv.abj7002

**Published:** 2022-02-18

**Authors:** Sushrut Kamerkar, Charan Leng, Olga Burenkova, Su Chul Jang, Christine McCoy, Kelvin Zhang, Kevin Dooley, Samuel Kasera, Tong Zi, Sílvia Sisó, William Dahlberg, Chang Ling Sia, Shil Patel, Karl Schmidt, Kyriakos Economides, Timothy Soos, Dalia Burzyn, Sriram Sathyanarayanan

**Affiliations:** Codiak BioSciences Inc., Cambridge, MA 02140, USA.

## Abstract

Effectiveness of checkpoint immunotherapy in cancer can be undermined by immunosuppressive tumor-associated macrophages (TAMs) with an M2 phenotype. Reprogramming TAMs toward a proinflammatory M1 phenotype is a novel approach to induce antitumor immunity. The M2 phenotype is controlled by key transcription factors such as signal transducer and activator of transcription 6 (STAT6), which have been “undruggable” selectively in TAMs. We describe an engineered exosome therapeutic candidate delivering an antisense oligonucleotide (ASO) targeting STAT6 (exoASO-STAT6), which selectively silences STAT6 expression in TAMs. In syngeneic models of colorectal cancer and hepatocellular carcinoma, exoASO-STAT6 monotherapy results in >90% tumor growth inhibition and 50 to 80% complete remissions. Administration of exoASO-STAT6 leads to induction of nitric oxide synthase 2 (*NOS2*), an M1 macrophage marker, resulting in remodeling of the tumor microenvironment and generation of a CD8 T cell–mediated adaptive immune response. Collectively, exoASO-STAT6 represents the first platform targeting transcription factors in TAMs in a highly selective manner.

## Teaser

ExoASO-STAT6 is a novel exosome therapeutic that selectively targets tumor macrophages, resulting in potent antitumoral activity.

## INTRODUCTION

The immune system plays an important protective function against tumor development and progression, effectively eliminating immunogenic cancer cells (*1*). Cancer immunotherapy with checkpoint inhibitors (CPIs) can induce long-lasting objective tumor responses in patients with metastatic cancers (*2*, *3*); however, ~85% of patients fail to benefit from these therapies, at least in part, because of immune evasion (*2*). The presence of myeloid cells in the tumor microenvironment (TME) constitutes one of the main mechanisms of resistance to CPI therapy (*2*, *4*, *5*). Tumor-associated macrophages (TAMs) are a major myeloid subset in the TME that exhibit a protumoral, immunosuppressive, “M2”-like phenotype. Abundant clinical data demonstrate a strong association of TAMs with poor prognosis (*5*–*7*). TAMs hamper antitumoral T cell responses through several mechanisms, including inhibition of T cell proliferation, blockade of CD8 T cell migration to the tumor, and recruitment of other immunosuppressive cells such as regulatory T cells (T_regs_) (*8*) into the TME. Targeting the protumoral M2 macrophages to relieve immune suppression and promote immune-mediated tumor regression is of key interest in cancer therapy.

Most therapeutic strategies targeting macrophages focus on blocking the recruitment of immature myeloid cells to the TME or attempting to reprogram TAMs into immune stimulatory M1-like macrophages. Several small-molecule drugs and monoclonal antibodies that target the recruitment and expansion of myeloid cells by inhibiting chemokines [e.g., CCL2 (C-C Motif Chemokine Ligand 2)] or growth factors [e.g., colony-stimulating factor 1 (CSF1)/CSF1 receptor (CSF1R)] (*5*) have not demonstrated substantial single-agent activity in preclinical models or clinical studies and require combination strategies (*5*, *9*, *10*). Similarly, modulation of TAM function via TREM2 (Triggering Receptor Expressed On Myeloid Cells 2) inhibition (*11*) or small-molecule inhibitors of phosphatidylinositol 3-kinase γ (PI3Kγ) also relies on combination with CPI to demonstrate tumor growth regressions in preclinical models (*12*). Other approaches such as CD40 agonists and cytokines like interferon-γ (IFN-γ) can induce macrophage reprogramming, but their therapeutic response has been restricted by dose-limiting toxicities (*13*, *14*).

Transcription factors that control macrophage M2 polarization are attractive targets for TAM reprogramming, including signal transducer and activator of transcription 6 (STAT6), STAT3, and CCAAT/enhancer binding protein β (C/EBPβ) (*15*). Given their broad expression profile, target cell selectivity is a critical aspect for effectively drugging these transcription factors. Small interfering RNA (siRNA) and antisense oligonucleotides (ASOs) are promising approaches for drugging transcriptional networks; however, they face limitations such as the lack of target cell selectivity, low cell permeability, and off-target related systemic toxicities, resulting in a narrow therapeutic window. Clinical development of an ASO targeting STAT3 has been limited by dose-limiting toxicities such as thrombocytopenia (*16*, *17*).

Exosome-based delivery systems hold the promise of targeted delivery to specific cell populations to expand the therapeutic index (*18*). Exosomes are extracellular vesicles of 30- to 200-nm diameter released by all cells (*19*). The observation that exosomes can transfer functional RNA and protein to recipient cells and mediate cell-to-cell communication locally and between organs has spurred translational research focused on developing exosome-based therapeutics. Exosomes can be engineered to deliver diverse therapeutic payloads to a desired target cell and are emerging as an efficient delivery system (*13*, *20*). The natural composition of the exosome lipid bilayer and the expression of several surface glycoproteins including prostaglandin F2 receptor negative regulator (PTGFRN) have been shown to enhance delivery of drug cargo to myeloid cells including TAMs (*20*, *21*).

STAT6 is a key regulator of the macrophage M2 transcriptional program in various pathological conditions, including cancer (*22*). Upon interleukin-4 (IL-4) or IL-13 stimulation, phosphorylated STAT6 dimerizes and translocates to the nucleus, where it induces the transcription of M2 signature genes such as *Arg1* (arginase 1), *Ccl17* (TARC), and *Mrc1* (Mannose Receptor C-Type 1) and represses the activation of M1 or inflammatory genes such as *Nlrp3*, *Ccl5*, and *Nos2* (*23*, *24*). In the TME, high levels of IL-4 produced by CD4 T cells, tumor cells, and other cell types drive the immunosuppressive phenotype of TAMs through the STAT6 pathway (*25*–*27*). TAMs from *Stat6*^−/−^ tumor-bearing mice display an M1 phenotype and demonstrate enhanced rejection of various tumor types (*28*–*30*), consistent with the role of STAT6 in polarizing TAMs to an M2 phenotype. Thus, STAT6 down-regulation in TAMs could be an effective approach to reprogram TAMs.

The studies reported here describe a unique exosome-based approach to reprogram TAMs to a proinflammatory M1 phenotype by selectively delivering a STAT6-targeting ASO to TAMs. This novel engineered exosome, exoASO-STAT6, demonstrates maximal biodistribution and STAT6-silencing activity in the liver after intravenous administration, with minimal effects in other tissues. exoASO-STAT6 shows robust antitumor activity as a monotherapy in multiple preclinical tumor models by inducing remodeling of the TME. Intratumoral or intravenous administration of exoASO-STAT6 in syngeneic tumor models of colorectal cancer and hepatocellular carcinoma (HCC), respectively, resulted in substantial tumor growth inhibition and complete tumor remission (CR) in most of the treated animals. In contrast, anti–programmed cell death protein 1 (PD-1) or anti-CSF1R antibodies failed to elicit substantial antitumor responses as monotherapy in these models. Immunophenotyping studies revealed a marked reduction in M2 macrophages in the tumors of exoASO-STAT6–treated animals that was associated with the appearance of M1 markers and accompanied by activation of a CD8 T cell–dependent adaptive immune response. We believe that exoASO-STAT6 is a novel first-in-class therapy that effectively reprograms TAMs and can markedly enhance antitumor immune responses.

## RESULTS

### Designing exosomes to mediate ASO delivery to macrophages

**Fig. 1.**
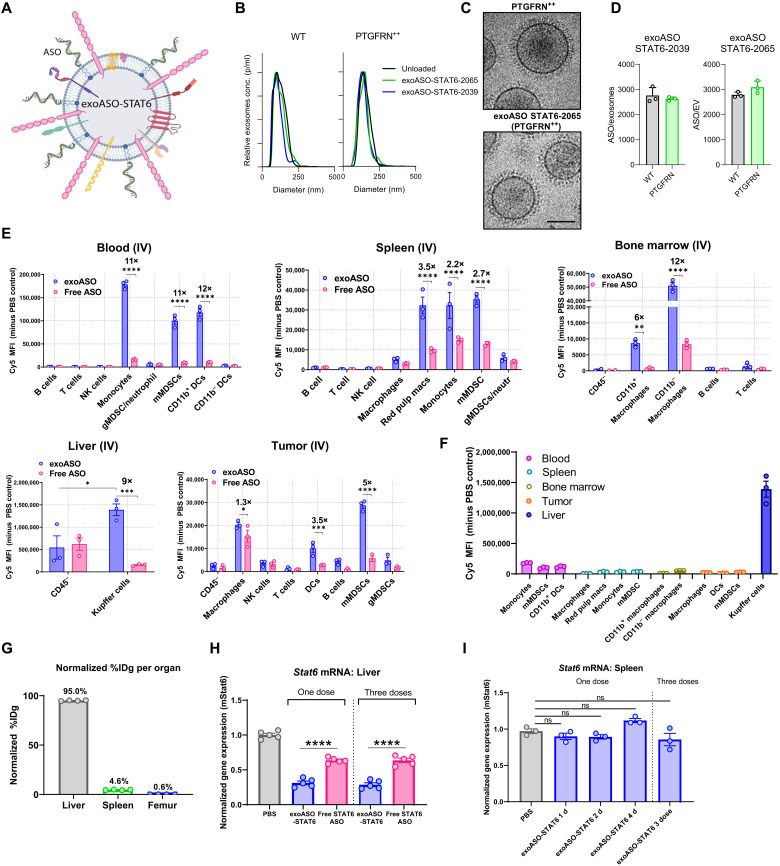
Exosome-mediated preferential delivery of ASOs to myeloid cells in vivo. (**A**) Schematic of STAT6 ASO loaded on PTGFRN^++^ exosomes. (**B**) Representative size distribution of WT and PTGFRN^++^ exosomes; unloaded or loaded with STAT6 ASO-2039 and ASO-2065, as measured by nanoparticle tracking analysis. (**C**) Representative cryogenic electron microscopy image of PTGFRN^++^ exosome; unloaded or loaded with STAT6 ASO-2065. (**D**) Quantification of loading (ASO/exosome) of STAT6 ASO-2039 and ASO-2065 on WT and PTGFRN^++^ exosomes. (**E**) In vivo distribution of Cy5-labeled exoASO STAT6-2039 (WT) as compared to free ASO. One hour after single intravenous (IV) dose (8 μg) of either exoASO or free STAT6-2039 (Cy5), mean fluorescence intensity (MFI) of Cy5 in the indicated immune cells and tissues of BALB/c mice bearing subcutaneous CT26 tumors is plotted. (**F**) Comparative analysis of the MFI of Cy5 (exoASO administered only) from (E), in the indicated myeloid cell populations from the indicated tissues. (**G**) Normalized % injected dose per gram (%IDg) as measured by positron emission tomography of C57Bl/6 mice injected intravenously with zirconium-89–labeled exosomes (PTGFRN^++^), %IDg was calculated at 55 min after single intravenous dose. gMDSC, granulocytic MDSC; mMDSC, monocytic MDSC. (**H**) Normalized gene expression analysis of changes in *Stat6* mRNA expression in the liver of naïve C57Bl/6 mice injected once or three times (TIW) intravenously with exoASO STAT6-2039 (PTGFRN^++^) (12 μg) or free STAT6 ASO-2039 (12 μg). (**I**) Normalized gene expression analysis of *Stat6* mRNA expression in the spleen of naïve C57Bl/6 mice injected once or three times (TIW) intravenously with PBS or exoASO STAT6-2039 (PTGFRN^++^) (12 μg). TIW, three times a week. Data are means ± SD (D) and ± SEM (E to I). *P < 0.05, **P < 0.01, ***P < 0.001, and ****P < 0.0001; ns, not significant. Two-way analysis of variance (ANOVA) with Sidak’s multiple comparisons test (E), and one-way ANOVA with Tukey’s multiple comparisons test (H and I).

Exosomes were generated using our engEx platform described recently by Dooley *et al.* (*20*). Previous studies with exosomes produced with this platform have demonstrated a natural tropism for macrophages and, particularly, M2 macrophages in vitro (*21*). We hypothesized that exosomes with these properties might preferentially deliver ASO to TAMs because of this tropism and effectively modulate gene expression preferentially in macrophages in the TME. To test this hypothesis, we developed exoASO-STAT6, an exosome loaded with an ASO targeting the STAT6 transcription factor (Fig. 1A). Exosomes [wild-type (WT) or overexpressing PTGFRN (PTGFRN^++^ exosomes)] were stringently and reproducibly purified from human embryonic kidney (HEK) 293 cells (Fig. 1, B and C, and fig. S1, A and C) (*20*). PTGFRN^++^ exosomes are being tested in clinical trials (ClinicalTrials.gov: NCT04592484) and are amenable for large-scale manufacturing and quality attributes required to support clinical translation of exoASO-STAT6. PTGFRN is the most abundant exosome surface protein naturally expressed in our WT exosomes (*20*); therefore, WT exosomes express PTGFRN, albeit at lower copy number than PTGFRN^++^ exosomes (fig. S1C) (*20*). Both WT and PTGFRN^++^ exosomes could effectively load STAT6 ASO on the surface, with no differences observed in exoASO-STAT6 biophysical properties, potency, and pharmacodynamic effects (Fig. 1, B to D, and figs. S1B, S2A, and S3I) and thus were used interchangeably throughout this work. For silencing STAT6, we identified two different ASOs: ASO-2039, a human/mouse cross-reactive sequence, and ASO-2065, a human-specific sequence. Transfection of a human cell line with either ASO to rule out hybridization off-target effects on related STAT family members showed STAT6-specific activity and no reduction in the mRNA levels of any of the genes tested (fig. S1D). The addition of a hydrophobic cholesterol tag and a linker enabled efficient exosome loading, resulting in >2000 copies per exosome with equal loading onto WT or PTGFRN^++^ exosomes (Fig. 1D).

To determine the cellular tropism of exoASO in vivo, the biodistribution of Cy5-labeled exoASO-STAT6 or free ASO was evaluated in CT26 colon cancer tumor–bearing mice. After intratumoral administration, minimal ASO-Cy5 signal was observed in the nonimmune compartment (CD45-negative cells), while TAMs showed the highest mean fluorescence intensity (fig. S1E). Other cells of myeloid origin [dendritic cells (DCs) and myeloid-derived suppressor cells (MDSCs)] showed intermediate levels of ASO signal. Similarly, 1 hour after intravenous administration, myeloid cells of the monocytic lineage (monocytes, macrophages, liver Kupffer cells, MDSCs, and myeloid DCs) showed the highest ASO-Cy5 signal. In contrast, B cells, T cells, natural killer (NK) cells, and granulocytic myeloid cells showed minimal association with exoASO. The exoASO-treated group consistently demonstrated significantly higher Cy5 signal compared to the free-ASO group in all tissues analyzed, including tumors (Fig. 1E). In blood monocytes, exosomes enhanced ASO delivery by 11-fold, compared to free ASO. A similar trend was observed in tissue macrophages (3.5-, 9-, and 12-fold increase versus free ASO for spleen red pulp macrophages, liver Kupffer cells, and bone marrow macrophages, respectively). Exosomes were also more effective in delivering ASO to myeloid cells in the CT26 subcutaneous tumors (1.3-, 3.5-, and 5-fold increase versus free ASO for TAMs, DCs, and MDSCs, respectively). These data demonstrate that exosomes potentiate the delivery of ASO to macrophages, monocytes, and MDSCs in the liver, peripheral blood, bone marrow, and TME.

A comparison of the fluorescence intensity among the major cell populations associated with ASO in each tissue clearly demonstrated that liver Kupffer cells display significantly higher ASO signal than any of the other subsets (8-, 27-, 43-, and 69-fold higher than the signal in blood, bone marrow, spleen, and subcutaneous tumor macrophages/monocytes, respectively) (Fig. 1F). This observation is in line with the result of a tissue/organ biodistribution study using ^89^Zr-labeled PTGFRN^++^ exosomes, which showed that 95% of the injected intravenous dose localized in the liver, while only 4.6 and 0.6% accumulated in the spleen and femur, respectively (Fig. 1G). In agreement with its tissue biodistribution pattern, exoASO-STAT6 induced potent *Stat6* knockdown in the liver of naïve mice when dosed intravenously [69% reduction in *Stat6* mRNA levels compared to phosphate-buffered saline (PBS) control], while no changes in *Stat6* expression were observed in the spleen (Fig. 1, H and I, and fig. S1F). Equivalent dose of free STAT6 ASO resulted in only a modest STAT6 knockdown (38%) in the liver, demonstrating that exosome-mediated delivery enhances ASO potency and enables preferential delivery to macrophages.

To investigate cellular uptake and intracellular trafficking of exoASO-STAT6, we evaluated exoASO uptake in M1- and M2-polarized human monocyte-derived macrophages (MDMs). To monitor phagocytosis and endocytosis, we labeled exoASO-STAT6 with pHrodo dye, a pH-sensitive dye that fluoresces in acidic environments such as the endolysosome compartment, and followed treated macrophages for 72 hours by live cell imaging. M2 macrophages showed higher fluorescence accumulation as compared with M1 macrophages (fourfold, fig. S1G), demonstrating preferential uptake of exoASO by M2-polarized macrophages. exoASO uptake by M2 macrophages was evaluated in the presence of the phagocytosis inhibitor cytochalasin D, the class A scavenger receptor inhibitor fucoidan, and the nonselective scavenger receptor inhibitor poly(I). Cytochalasin D treatment resulted in maximal inhibition (93%) of uptake, whereas treatment with fucoidan and poly(I) blocked ~50% uptake (fig. S1H). Consistent with the previously reported low expression of scavenger receptors in M1 macrophages (*31*), fucoidan did not inhibit uptake in these cells, while treatment with poly(I) resulted in only 30% inhibition (fig. S1H). Thus, exoASO uptake is superior in M2 macrophages and is mediated by phagocytosis and scavenger receptor–mediated mechanisms.

### Effective reprogramming of immunosuppressive M2 macrophages to proinflammatory M1 macrophages in vitro

**Fig. 2.**
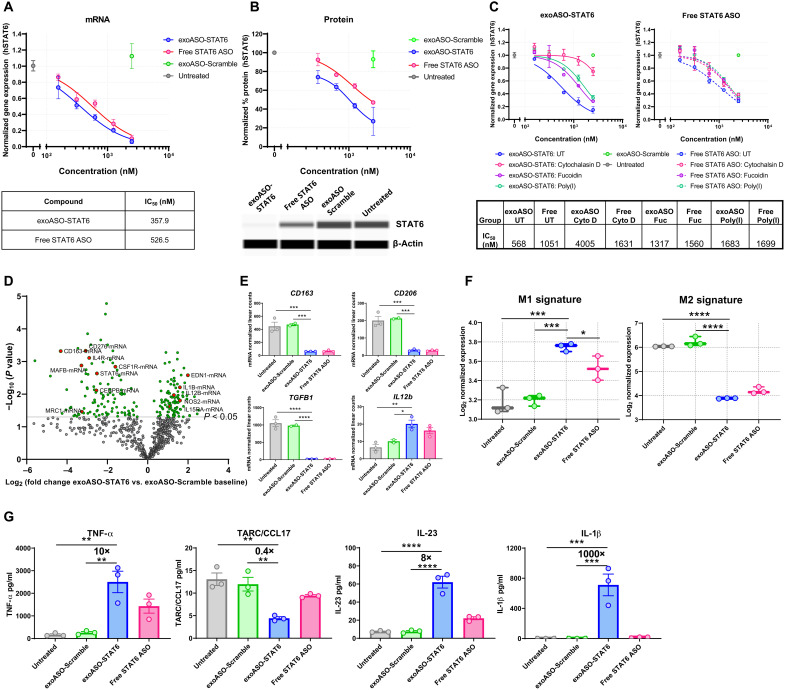
Persistent reduction of STAT6 expression by exoASO leads to reprogramming of M2 macrophages. (**A**) Normalized gene expression analysis by reverse transcription quantitative polymerase chain reaction (RT-qPCR) showing knockdown of STAT6 mRNA expression in M2 MDMs treated for 48 hours with either exoASO STAT6-2065 (PTGFRN^++^), free STAT6 ASO-2065, or exoASO-Scramble. hSTAT6, human STAT6; IC_50_, median inhibitory concentration. (**B**) Reduction in protein expression of STAT6 as measured by whole-exome sequencing, in M2 MDMs treated for 96 hours with either exoASO STAT6-2065 (PTGFRN^++^), free STAT6 ASO-2065, or exoASO-Scramble, normalized to housekeeping gene β-actin. (**C**) Normalized gene expression analysis by RT-qPCR showing knockdown of STAT6 mRNA expression in M2 MDMs untreated (UT) or pretreated with either 10 μM cytochalasin D, poly(I) (10 μg/ml), or fucoidan (500 μg/ml). M2 MDMs were then treated with exoASO STAT6-2039 (PTGFRN^++^), free STAT6 ASO-2039, or exoASO-Scramble for 48 hours. (**D**) NanoString gene expression analysis as depicted by a volcano plot of changes in gene expression of exoASO-STAT6 versus exoASO-Scramble baseline and of M2 MDMs treated for 48 hours with 2.5 μM exoASO STAT6-2039 (WT), free STAT6 ASO-2039, or exoASO-Scramble. One representative donor of three is shown. (**E**) Modulation of expression levels of *CD163*, *CD206*, *TGFB1*, and *IL12b* from (D). (**F**) M1 and M2 signature analysis (*31*), calculated from gene expression analysis from (D). (**G**) Cytokine analysis depicting modulation of TNF-α, CCL17, IL-23, and IL-1β using a multiplex flow cytometry assay and of M2 MDMs treated for 48 hours (24 hours with LPS) with 2.5 μM of either exoASO STAT6-2039 (WT), free STAT6 ASO-2039, or exoASO-Scramble. One representative donor of four is shown. Data are means ± SD. *P < 0.05, **P < 0.01, ***P < 0.001, and ****P < 0.0001. One-way ANOVA with Tukey’s multiple comparisons test (E to G).

We next evaluated the potential of exoASO-STAT6 to reduce *STAT6* mRNA and protein expression in human M2-polarized MDMs and to induce reprogramming to an M1 phenotype. M2-polarized MDMs were treated with exoASO-STAT6, exosomes loaded with a control exoASO (exoASO-Scramble), or free ASO as indicated. exoASO-STAT6 treatment (48 hours) reduced *STAT6* mRNA expression in a dose-dependent manner (Fig. 2A) and was about twofold more potent than the free ASO in silencing *STAT6* mRNA expression (Fig. 2A). No *STAT6* silencing was observed in the control exoASO-treated macrophages, and no differences were observed between WT and PTGFRN^++^ exoASO-STAT6 (fig. S2A). mRNA reduction was durable, resulting in 80% reduction for at least 5 days (fig. S2B). Consistent with the mRNA reduction, a dose-dependent reduction in STAT6 protein was also observed at 96 hours, with exoASO-STAT6 being more effective than free ASO (~75% reduction versus 45% reduction) (Fig. 2B). The durability of STAT6 protein reduction after 1-day treatment with exoASO-STAT6 was at least 14 days, the last time point evaluated (fig. S2C). We compared *STAT6* mRNA silencing by exoASO-STAT6 and free STAT6 ASO in the presence of phagocytosis and scavenger receptor inhibitors. In line with the effects observed in uptake (fig. S1H), treatment with fucoidan and poly(I) partially reduced [2.3- and 3-fold increase in median inhibitory concentration (IC_50_), respectively] the potency of exoASO-STAT6, while cytochalasin D treatment markedly reduced (7-fold increase in IC_50_) the potency of exoASO-STAT6 treatment (Fig. 2C). In the free STAT6 ASO–treated group, there were no significant changes in the *STAT6* mRNA silencing, following inhibitor treatment (Fig. 2C). These results show an important distinction between free ASO and exoASO in the uptake mechanism by macrophages, which might underlie the difference in the potency of these two compounds.

To investigate whether the reduction in STAT6 expression by exoASO-STAT6 was sufficient to induce M2 to M1 reprogramming, gene expression changes were measured in human M2-polarized macrophages treated with exoASO-STAT6 NanoString analysis. exoASO-STAT6 treatment resulted in significant reduction of M2 genes [e.g., *CD206*, *CD163*, transforming growth factor–β (*TGFB1*), *CSF1R*, and *CEBPB*] and concomitant increase in proinflammatory genes such as *IL12B*, *IL1B*, and nitric oxide synthase 2 (*NOS2*) (Fig. 2, D and E). In addition, STAT6 pathway genes [e.g., *IL4R*, *STAT6*, *MRC1* (Mannose Receptor C-Type 1), and *MAFB* (MAF BZIP Transcription Factor B)] (*23*, *32*–*34*) were also significantly reduced (Fig. 2D). We compared the gene signature of M2 macrophages treated with exoASO-STAT6 with reported canonical M1 and M2 macrophage gene expression signatures (*31*). exoASO-STAT6 treatment resulted in significant (*P* = 0.0004) induction of a M1 macrophage gene signature and fivefold reduction of a M2 macrophage gene signature (*P* < 0.0001) compared to macrophages treated with control exoASO (Fig. 2F and fig. S2D). Control exoASO treatment did not result in measurable reduction of M2 signature genes or increase in M1 signature genes. Free STAT6 ASO treatment resulted in macrophage reprogramming although to a lesser degree as compared to the dose-matched exoASO-STAT6 (Fig. 2F). Cytokine production in M2-polarized MDMs was analyzed after 24-hour treatment with lipopolysaccharide (LPS). exoASO-STAT6 induced multiple M1 cytokines tumor necrosis factor–α (TNF-α) (16-fold), IL-23 (7-fold), IL-12 (10-fold), and IL-1β (150-fold) and a 4-fold decrease in M2 chemokine (CCL17), compared with control exoASO (Fig. 2G and fig. S2, F and G). The induction of M1 cytokines and reduction of M2 cytokines in most cases were more pronounced in the exoASO-STAT6–treated group as compared to free STAT6 ASO (Fig. 2G and fig. S2G). These data establish that exoASO-STAT6 skews suppressive macrophages toward a proinflammatory phenotype. Both exoASO-STAT6-2039 and exoASO-STAT6-2065 were effective in inducing STAT6 silencing and effective reprogramming in M2 MDMs, ruling out a sequence-specific effect (fig. S2, H and I).

### exoASO-STAT6 induces potent single-agent antitumor activity in CT26 tumor model following intratumoral administration

Colorectal carcinoma is enriched in TAMs and nonresponsive to anti–PD-1 or anti–programmed death-ligand 1 targeting therapies (*35*). The antitumor activity of exoASO-STAT6 was evaluated in an aggressive colorectal cancer (CT26) syngeneic tumor model. This model is enriched in TAMs (*36*) and shows limited response to checkpoint therapies, including anti–PD-1, and therefore is representative of a TAM-rich checkpoint refractory tumor model. Previous studies with this model show limited tumor growth inhibition to other macrophage-targeted therapies including anti-CSF1R antibodies (*37*–*39*).

**Fig. 3.**
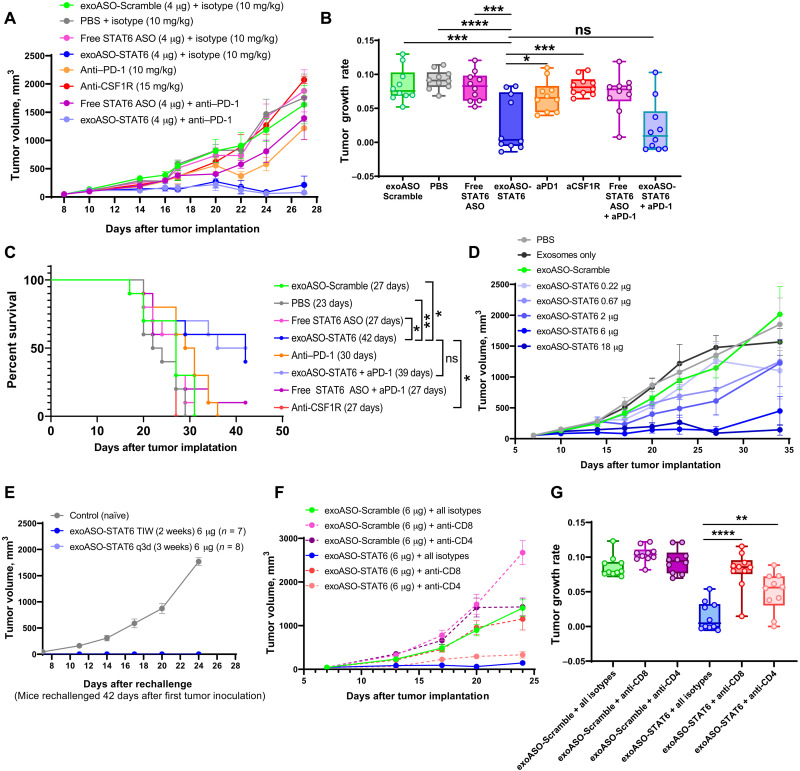
exoASO-STAT6 treatment results CD8 T cell–dependent monotherapy efficacy in CT26. (**A**) Tumor growth volumes of BALB/c mice bearing subcutaneous CT26 tumors, injected intratumorally (TIW) with PBS, exoASO-Scramble (4 μg), free STAT6 ASO-2039 (4 μg), and exoASO STAT6-2039 (WT) (4 μg); intraperitoneally [twice a week (BIW)] with anti–PD-1 monoclonal antibody (10 mg/kg) and anti-CSF1R (15 mg/kg); and a combination of exoASO STAT6-2039 (WT) (4 μg) or free STAT6 ASO-2039 (4 μg) with anti–PD-1 monoclonal antibody (10 mg/kg); *n* = 10 mice per group. (**B**) Tumor growth rates from data in (A). (**C**) Kaplan-Meier survival curve analysis of data from CT26 mice in (A), log-rank Mantel-Cox test. (**D**) Tumor growth volumes of BALB/c mice bearing subcutaneous CT26 tumors, injected intratumorally with PBS, exosomes only, exoASO-Scramble (6 μg) (BIW, 3 weeks), or exoASO STAT6-2039 (PTGFRN^++^) (0.22, 0.67, 2, 6, and 18 μg) (BIW, 3 weeks); *n* = 10 mice per group. (**E**) Tumor growth volumes of mice rechallenged with CT26 tumors on the opposite flank of complete responders from (fig. S3D), Naïve BALB/c mice bearing subcutaneous CT26 tumors were used as controls; *n* = 10 mice for control group. (**F**) Tumor growth volumes of BALB/c mice bearing subcutaneous CT26 tumors after CD8 and CD4 T cell depletion, injected intratumorally with either exoASO-Scramble (6 μg) (TIW, 2 weeks) or exoASO STAT6-2039 (PTGFRN^++^) (6 μg) (TIW, 2 weeks). Mice received one dose of anti-CD8 or anti-CD4 antibody (10 mg/kg) or of isotype control antibody (10 mg/kg), before intratumoral injections, and BIW thereafter; *n* = 10 mice per group. (**G**) Tumor growth rates from data in (F). Data are means ± SEM. *P < 0.05, **P < 0.01, ***P < 0.001, and ****P < 0.000. One-way ANOVA with Tukey’s multiple comparisons test (B and G).

We investigated the efficacy of exoASO-STAT6 in the CT26 model following intratumoral injection to maximize delivery of drug to the tumor. Animals were dosed three times a week (TIW) with exoASO-STAT6 (4 μg) or equal amounts of control exoASO or free STAT6 ASO for 2 weeks. For comparison, anti–PD-1 or anti-CSF1R antibody therapies were administered intraperitoneally. Complete tumor remission (CRs) was observed in 6 of 10 mice treated with exoASO-STAT6 (Fig. 3, A and B, and fig. S3A). The antitumoral activity of exoASO-STAT6 was comparable when WT or PTGFRN^++^ exosomes were used (fig. S3I). In contrast, no CRs were observed with free STAT6 ASO or control exoASO. Consistent with previous publications, anti-CSF1R monotherapy failed to inhibit tumor growth. Anti–PD-1 therapy resulted in modest growth inhibition with no significant reduction in tumor growth rate, and no CRs were observed. Combination of anti–PD-1 with exoASO-STAT6 did not show any additive or synergistic effects in antitumoral efficacy (Fig. 3, A and B, and fig. S3A). exoASO-STAT6 monotherapy and combination treatments significantly prolonged the survival by day 42 (*P* = 0.0073 versus PBS group) and day 39 (*P* = 0.0041 versus PBS group), respectively (Fig. 3C). Intravenous dosing of exoASO-STAT6 in this model did not result in profound monotherapy activity. When dosed intravenously, exoASO-STAT6 resulted in modest tumor growth inhibition (fig. S3, B and C). Comparison of the biodistribution of exoASO after intravenous or intratumoral administration demonstrated that intratumoral administration resulted in maximal exoASO delivery to the TAMs in subcutaneous CT26 tumors (fig. S3C). These results suggest that maximal targeting of TAM is required for optimal exoASO-STAT6 efficacy.

To optimize the dose and dose regimen of exoASO-STAT6, we compared the efficacy of TIW dosing for 2 weeks to twice a week (BIW) dosing for 3 weeks. Both doses resulted in comparable tumor growth inhibition resulting in 7 of 10 and 8 of 10 CRs, respectively (fig. S3, D and E). The less frequent dosing was chosen on the basis of the duration of *Stat6* knockdown in tumor CD11b cells, which peaks at 48 hours following intratumoral dosing (fig. S3F). Next, we evaluated the dose response from 0.22 to 18 μg per dose of exoASO-STAT6 in the CT26 model. A dose-dependent decrease in tumor volume of the injected tumor was observed following treatment with exoASO-STAT6 (Fig. 3D). Consistent with previous studies, empty exosomes (*21*) or control ASO–containing exosomes failed to control tumor growth. Minimal tumor growth inhibition was observed at the 0.2-μg dose. At intermediate dose levels (0.6 and 2 μg BIW), CR was observed in 10% of the animals. At the higher dose levels (6 or 18 μg BIW), CR was observed in 50 and 60% of the animals, respectively, with a significant reduction in tumor growth rate (*P* < 0.001) (fig. S3G).

Tumor rechallenge studies were also carried out in the CT26 model to assess the development of immunological memory in animals treated with effective doses of exoASO-STAT6. On day 42 after the primary tumor cell inoculation, animals with a CR to the initial tumor (fig. S3, D and E) were rechallenged with an inoculum of CT26 cells in the opposing flank. Mice that achieved a CR from the primary tumor uniformly rejected growth of the secondary CT26 cells. In contrast, uniform tumor growth was observed in naïve mice (Fig. 3E).

The requirement for CD4^+^ and CD8^+^ T cells in mediating antitumor responses was further characterized with antibody depletion studies. Robust tumor growth inhibition (*P* < 0.0001) was observed in the exoASO-STAT6–treated isotype control group, resulting in CR in 60% CR (Fig. 3, F and G, and fig. S3H). Depletion of CD4 T cells only partially inhibited the effect of exoASO-STAT6 on tumor growth (Fig. 3, F and G). In contrast, no CR was observed in the anti-CD8 antibody–treated group, and the tumor growth rate was comparable to control groups (Fig. 3G). These data demonstrate the critical role played by CD8^+^ T cells in mediating exoASO-STAT6 antitumor immunity. This study represents the first macrophage-targeted therapy that demonstrates substantial tumor growth inhibition, resulting in complete tumor remission when evaluated as a single agent.

### Effective remodeling of the TME by exoASO-STAT6 in the CT26 tumor model

**Fig. 4.**
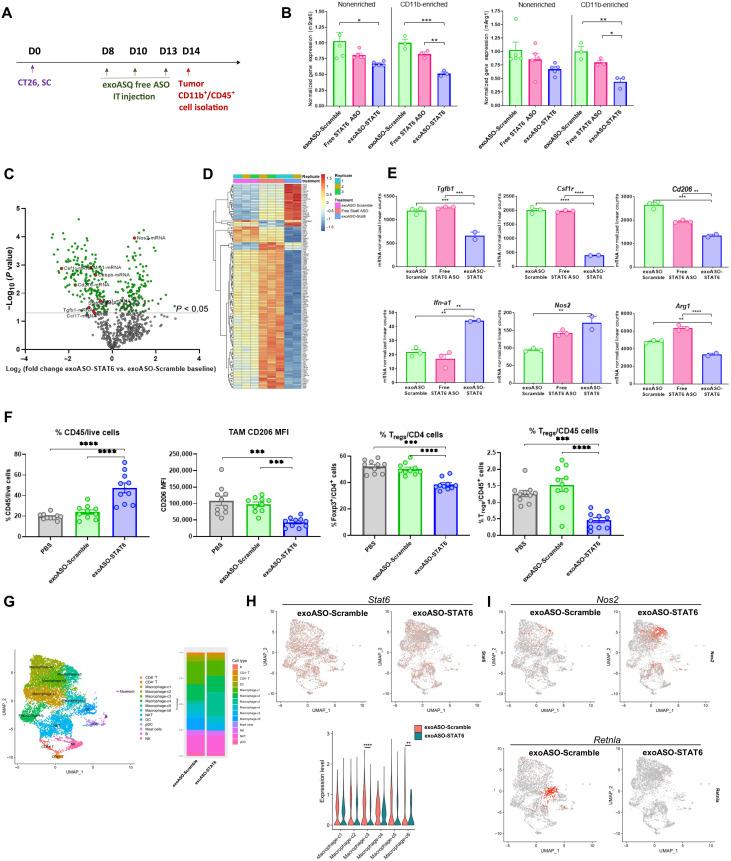
Effective reprogramming of TAMs by exoASO-STAT6 results in remodeling of TME. (**A**) Schematic of dosing schedule for (B) to (E) and (G) to (I). BALB/c mice bearing subcutaneous (SC) CT26 tumors injected with exoASO-Scramble (4 μg), exoASO STAT6-2039 (WT) (4 μg), or free STAT6 ASO-2039 (4 μg). IT, intratumoral. (**B**) Normalized gene expression analysis by RT-qPCR of changes in *Stat6* and *Arg1* mRNA expression from whole tumors and CD11b-enriched fractions. (**C**) NanoString gene expression analysis as depicted by a volcano plot of changes in gene expression of exoASO-STAT6 versus exoASO Scramble baseline, from CD11b-enriched fractions from (B). (**D**) Heatmap of common differentially expressed genes from all groups from (C). (**E**) Graphical representation of changes in expression levels of *Tgfb1*, *Csf1r*, *CD206*, *Ifn-a1*, *Nos2*, and *Arg1* from (C). (**F**) Flow cytometry analysis of % of total immune cells, and MFI of (CD206^+^) TAMs and % of T_regs_ (FoxP3^+^) within CD4^+^ or CD45^+^ immune cell population from tumors of BALB/c mice bearing subcutaneous CT26 tumors, injected intratumorally with PBS, exoASO-Scramble (6 μg) (TIW, 1 week), or exoASO STAT6-2039 (PTGFRN^++^) (6 μg) (TIW, 1 week); *n* = 10 mice per group. (**G**) UMAP plot from scRNA-seq of intratumoral cells of all groups merged to identify individual immune cell populations. BALB/c mice bearing subcutaneous CT26 tumors were injected intratumorally with exoASO-Scramble (6 μg) or exoASO STAT6-2039 (PTGFRN^++^) (6 μg) (TIW, 1 week). (**H**) UMAP plots from data from (G), showing global changes in expression and quantification of *Stat6* in immune cell populations. (**I**) UMAP plots from data from (G), showing global changes in expression of *Nos2* and *Retnla* (*Fizz1*) in immune cell populations. Data are means ± SEM. *P < 0.05, **P < 0.01, ***P < 0.001, and ****P < 0.000. One-way ANOVA with Tukey’s multiple comparisons test (B, E, F, and H).

The pharmacodynamic effect of exoASO-STAT6 treatment was investigated in the CT26 tumor model. CT26 tumor–bearing mice were treated with three intratumoral doses of exoASO-STAT6 (4 μg) or controls as indicated in Fig. 4A. *Stat6* expression was analyzed in tumor CD11b cells enriched by positive cell sorting (fig. S4A). Treatment with exoASO-STAT6 induced about a 50% reduction of *Stat6* mRNA in the CD11b-enriched cells, while only 17% knockdown was observed after treatment with free STAT6 ASO (Fig. 4B). Moreover, enriched CD11b cells showed a 56% reduction in the expression of the M2 gene *Arg1*, whereas free STAT6 ASO–mediated reduction in *Arg1* expression was significantly less (20%) (Fig. 4B).

To assess in more detail the impact of Stat6 knockdown on the TME myeloid compartment, we performed gene expression analysis using the NanoString platform in the pool of enriched tumor CD11b cells. Macrophage-expressed genes showed clear changes, consistent with the in vitro data described above (Fig. 2). Genes associated with M2 macrophage phenotype such as *Csf1r* were down-regulated by up to 80% in the exoASO-STAT6 treatment group as compared to the control exoASO group. Similarly, a twofold increase in M1-related genes such as *Nos2* was observed in the exoASO-STAT6 group, suggesting an effective in vivo reprogramming of TAMs following exoASO-STAT6 treatment (Fig. 4, C to E). An equivalent dose of free STAT6 ASO treatment did not result in measurable modulation of M2 or M1-related genes, demonstrating that exosome-mediated delivery is required in vivo to induce effective reprogramming of TAMs.

Next, we evaluated the effect of exoASO-STAT6 treatment on the composition of the tumor immune infiltrate. Immunophenotyping of CT26 tumors after intratumoral treatment with exoASO-STAT6 or control exoASO as described in Fig. 4A was performed to evaluate global changes in the immune cells from the TME. Cytofluorometric analyses of digested tumors showed a significant twofold increase in the immune infiltrate (% CD45^+^ cells) in the exoASO-STAT6 versus control exoASO group (Fig. 4F). Despite the increase in total immune cells, the frequency of total macrophages and total T cells in the tumor remained unchanged (fig. S4B). The expression of the M2 marker CD206 was reduced by 60% in F4/80^+^ TAMs by exoASO-STAT6 (Fig. 4F), supporting the results from the gene expression analysis (Fig. 4E). Analysis of the T cell infiltrate showed no changes in the frequency of CD8 T cells (fig. S4B). However, a significant decrease (*P* < 0.0001) in the proportion of T_regs_ within the CD4 compartment was observed as well as a 3.3-fold reduction in the frequency of T_regs_ in the immune infiltrate (Fig. 4F).

Assessment of gene expression of tumor infiltrating cells by single-cell RNA sequencing (scRNA-seq) confirmed that exoASO-STAT6 causes significant changes in the monocyte/macrophage populations in the tumor (Fig. 4, G and H, and fig. S4, C and D). Unbiased analysis identified six monocyte/macrophage populations with distinct gene expression profile within the CT26 tumors (Fig. 4G and table S1). Clusters c3 and c6 showed significant reduction in *Stat6* expression induced by exoASO-STAT6 compared to the control exoASO treatment (*P* < 0.0001 and *P* < 0.01 for c3 and c6, respectively) (Fig. 4H). Two subsets, c3 and c5, expanded in response to exoASO-STAT6 (Fig. 4G and fig. S4, C and D). Levels of the M1 marker *Nos2* increased significantly in the exoASO-STAT6 treatment group, in particular in the expanded clusters c3 and c5 (Fig. 4I and table S2). *Nos2* induction has been associated with macrophage reprogramming and antitumor immune response (*13*, *40*, *41*). In contrast, clusters c1 and c6 were reduced by exoASO-STAT6 treatment (Fig. 4G and fig. S4, C and D). Cluster c6 is represented by cells expressing high levels of *Cd163* and *Retnla* (*Fizz1*), a population predominantly associated with M2-polarized macrophages (Fig. 4I and table S1) (*42*). It should be noted that *Fizz1* has been previously reported to be regulated by IL-4 and STAT6 signaling pathways (*43*). exoASO-STAT6 treatment profoundly reduced the levels of this population as compared to control exoASO. Cluster 1 is represented by high levels of *Cd206*, *Trem2* [a marker of protumorigenic TAMs (*11*)], and subcomponents of the complement subunit C1q, an important mediator of innate inflammation that is induced by M2-reprogramming cytokines like IL-4 (table S1) (*44*). These results confirm a reduction in the monocyte/macrophage cells with M2-polarized markers and an increase in the M1-polarized population. Furthermore, the scRNA-seq study also revealed that exoASO-STAT6 induced activation of the tumor-infiltrating CD8 T cells, which showed increased expression of effector/activation genes such as *Gzmb* and *Id2*, and decrease of exhaustion markers such as *Lag3* (fig. S4E). In summary, profound changes were observed in the macrophages and other infiltrating immune cells in CT26 tumors treated with exoASO-STAT6, resulting in the remodeling of the TME toward immune activation and antitumor immunity.

### Systemic administration of exoASO-STAT6 results in potent monotherapy antitumor response in an HCC model

**Fig. 5.**
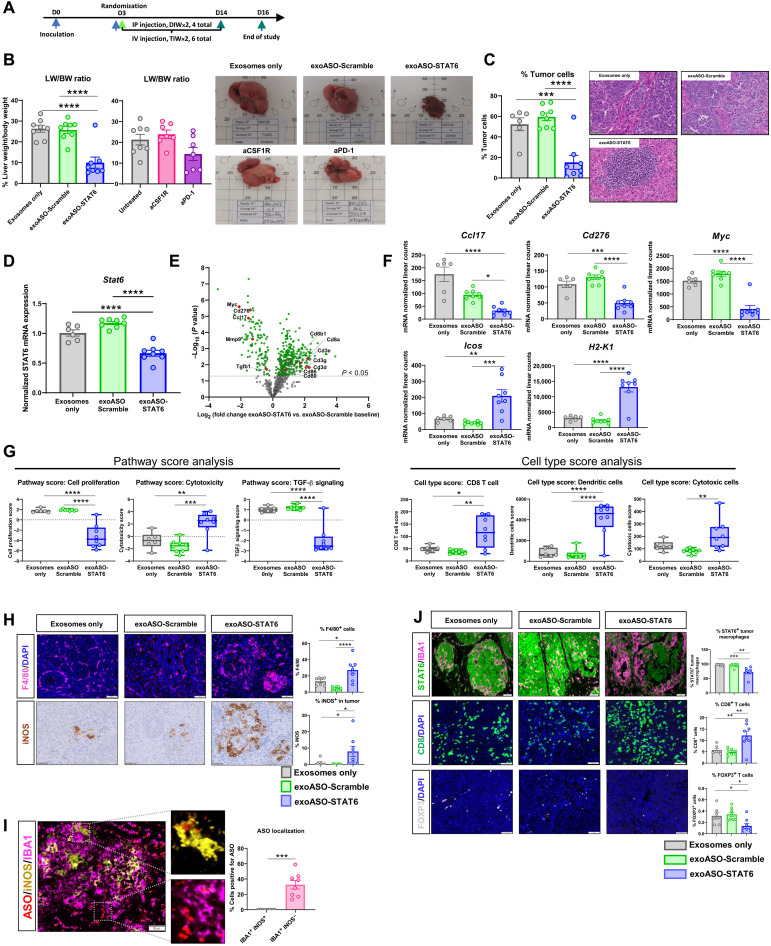
Systemic administration of exoASO-STAT6 results in a potent monotherapy antitumor response. (**A**) Schematic of dosing schedule for (B) to (J). IP, intraperitoneal. (**B**) Antitumoral efficacy as represented by liver weight (LW) versus body weight (BW) ratio of C57Bl/6 mice bearing orthotopic Hepa1-6 tumors in the liver, injected intravenously with exosomes only, exoASO-Scramble (12 μg) (TIW, 2 weeks), or exoASO STAT6-2039 (PTGFRN^++^) (12 μg) (TIW, 2 weeks) or intraperitoneally with anti-CSF1R (10 mg/kg) (DIW) or anti–PD-1 (10 mg/kg) (BIW). Representative gross images at study end point are shown. (**C**) Percentage (%) of tumor cells as calculated from hematoxylin and eosin–stained sections of livers from (B). (**D**) Normalized gene expression analysis of modulation of *Stat6* mRNA expression in whole Hepa1-6 tumor livers from (B). (**E**) NanoString gene expression analysis as depicted by a volcano plot of changes in gene expression of exoASO-STAT6 versus exoASO-Scramble baseline from (B). (**F**) Graphical representation of changes in expression levels of *Ccl17*, *Cd276*, *Myc*, *H2-K1*, and *Icos* from (E). (**G**) Pathway score analysis of data in (E) as calculated by nSolver Analysis Software. Cell type score profiling of data in (E) as calculated by nSolver Analysis Software. (**H**) Representative images and quantification of F4/80 (macrophage) and iNOS expression, performed by immunofluorescence and IHC (immunohistochemistry) respectively, in Hepa1-6 tumor sections from (B). (**I**) Representative images and quantification of ASO localization in iNOS-positive, IBA1^+^ (M1), and iNOS-negative, IBA1^+^ (M2), macrophages. (**J**) Representative images and quantification of STAT6 expression in macrophages (IBA1^+^) within the tumor, cytotoxic T cells (CD8^+^), and regulatory T cells (FOXP3^+^) performed by immunofluorescence in Hepa1-6 tumor sections from (B). Data are means ± SEM. *P < 0.05, **P < 0.01, ***P < 0.001, and ****P < 0.0001. One-way ANOVA with Tukey’s multiple comparisons test (B to D, F, G, and J), Sidak’s multiple comparisons test (H), and unpaired two-tailed Student’s *t* test (I). DAPI, 4′,6-diamidino-2-phenylindole.

HCC tumors are highly enriched in TAMs, and most patients do not benefit from anti–PD-1 therapy (*45*–*47*). The biodistribution of exosomes to the liver and macrophages in the tumor makes this an ideal cancer type for exoASO-STAT6. In addition, exosome-mediated delivery enhances ASO potency compared to free ASO (Fig. 1H and fig. S1H) also when dosed systemically, suggesting that tumors located in the liver can be targeted by intravenous administration of exoASO. To assess the antitumoral efficacy of systemically dosed exoASO-STAT6 in HCC, we used the Hepa1-6 orthotopic model (Fig. 5A) The treatment was initiated 3 days after tumor inoculation. This is a very aggressive tumor model that shows minimal response to anti–PD-1 therapy and is nonresponsive to other macrophage-depleting therapies such as anti-CSF1R (Fig. 5B).

ExoASO-STAT6 treatment resulted in a 62% reduction in tumor burden, measured as the ratio of liver to body weight. In contrast, the control exoASO–treated animals did not show any measurable decrease in tumor burden (Fig. 5B). Quantification of tumor cells in histological sections also showed a significant decrease (71%) in tumor burden in the exoASO-STAT6 treatment group compared to the exosome-only control (Fig. 5C and fig. S5A). Close examination of hematoxylin and eosin–stained liver sections and histopathological scoring of the tumor content showed markedly smaller tumor islands in the exoASO-STAT6–treated mice and conspicuous immune infiltrate colocalizing with tumor areas (Fig. 5C), with 50% of the animals presenting major pathological responses (tumor area < 10%), including one mouse with complete remission (tumor area ≤ 1%). Majority of exoASO-STAT6–treated mice, including those with residual tumor, showed heavy immune cell infiltration (fig. S5B). Combination therapy with an anti–PD-1 antibody resulted in a profound reduction in tumor burden (85% versus exoASO-Scramble). In contrast, exoASO-STAT6 monotherapy resulted in a 53% reduction in tumor burden, while anti–PD-1 alone resulted in a 9% increase in tumor burden as compared to exoASO-Scramble (fig. S5, C and D). Histopathological analysis revealed higher levels of immune cell infiltration in the combination group compared to the exoASO-STAT6 monotherapy group (fig. S5E). While 50% of the animals in the exoASO-STAT6 monotherapy group showed a high tumor inflammation score, the combination with anti–PD-1 increased the number of animals with high tumor inflammation to 87.5% (fig. S5E).

The effect of exoASO-STAT6 treatment on *Stat6* expression was evaluated in whole liver tissue by real-time reverse transcription quantitative polymerase chain reaction. Results showed that exoASO-STAT6 reduced Stat6 mRNA levels by 34% compared to the exosome-only–treated group, demonstrating effective target gene knockdown (Fig. 5D). Immunohistochemistry assays on total liver sections showed that exoASO-STAT6 induced 61% knockdown of STAT6 protein compared to baseline expression in the exosome-only group (fig. S5F). Because STAT6 protein was expressed by both tumor cells and macrophages, we specifically analyzed the expression of STAT6 in IBA1^+^ (Ionized Calcium-Binding Adapter Molecule 1) macrophages within the tumor areas. In the exoASO-STAT6–treated group, the percentage of STAT6-positive TAMs was significantly lower than in the control groups (Fig. 5J). To investigate the mechanism of action underlying the significant antitumoral activity of exoASO-STAT6 in this model, gene expression analysis was performed on whole liver tissue at the end of the study. The expression of a pan-cancer gene panel was assessed by NanoString. Treatment with exoASO-STAT6 induced significant changes in gene expression compared to exosome-only and control exoASO (Fig. 5, E and F). Notably, several genes associated with an antitumoral T cell response were exclusively up-regulated in the exoASO-STAT6 group [e.g., CD3e, CD8, ICOS (Inducible T Cell Costimulator), CD80, and CD86] (Fig. 5, E and F). In contrast, this group showed decreased expression of M2 genes such as Cd276, Myc, and Ccl17. Pathway and cell-type analysis confirmed not only the induction of a potent antitumoral immune response but also a decrease in tumor cell proliferation markers (Fig. 5G and fig. S5, G and H). Compared to both control groups, the exoASO-STAT6 group displayed significantly increased scores for cytotoxic cells, CD8 T cells, dendritic cells, and interferon signaling and significantly decreased scores for TGF-β signaling, cell proliferation, and angiogenesis. The changes in the TME were confirmed by immunohistochemistry on liver sections. Results showed an increase in macrophage infiltration and a concomitant increase in expression of the M1 macrophage marker inducible nitric oxide synthase (iNOS) in the exoASO-STAT6 group (2- and 8.5-fold compared to exosome-only group, respectively) (Fig. 5H). Moreover, the ASO colocalized preferentially with the iNOS-negative macrophages, confirming that exoASO-STAT6 also shows selective tropism to M2 macrophages compared to M1 (Fig. 5I). Last, a strong CD8 T cell infiltration (2.1-fold increase versus exosome-only group) and a significant reduction in T_reg_ infiltration (2.3-fold decrease versus exosome-only group) were observed (Fig. 5J).

Given the strong immune response elicited at the tumor site by intravenous dosing of exoASO-STAT6, we assessed whether systemic inflammation was also induced by exoASO treatment by measuring cytokines in the serum of treated animals at the end of the study. As shown in tables S3 and S4, no significant changes were observed between exoASO-STAT6 and control exoASO treatments, sham animals that did not have implanted tumors, or naïve mice. In addition, no body weight loss and no signs of hepatotoxicity were observed in any of the treatment groups as observed in the serum levels of aspartate aminotransferase and alanine aminotransferase (fig. S5, I and J). These data demonstrate significant antitumor activity with exoASO-STAT6 therapy in the Hepa1-6 orthotopic tumor model and a lack of systemic inflammation by intravenous dosing of exoASO-STAT6.

### STAT6 macrophage signature correlates with poor disease prognosis in HCC

**Fig. 6.**
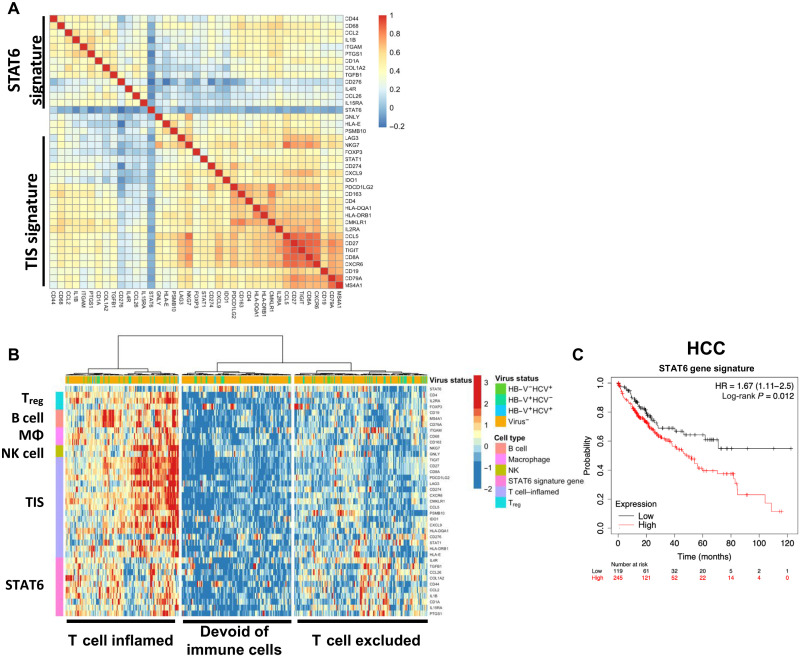
STAT6 macrophage signature correlates with poor disease prognosis. (**A**) Spearman’s correlation heatmap of genes within a unique STAT6 macrophage signature and tumor inflammation signature (TIS), depicting a subset of 10 genes that are coherently expressed across the dataset. (**B**) Heatmap based on a unique STAT6 macrophage gene signature, depicting identification of three molecular subsets based on gene signature changes across patients with HCC, using a panel of immune cell markers (T_regs_, B cells, macrophages, NK cells, and TIS) and a STAT6 macrophage gene signature. Data were generated using LIHC (Liver Hepatocellular Carcinoma) tumor samples from TCGA. Genes and samples have been hierarchically clustered on the basis of their gene expression pattern with Ward’s method. (**C**) Kaplan-Meier curves of overall survival probability in HCC, based on analysis of patients with high or low STAT6 macrophage gene signature. Results were generated using Kaplan-Meier plotter and log-rank Mantel-Cox test.

To investigate the translational significance of our preclinical findings, we focused our initial analysis in human HCC tumors. We generated a STAT6 signature based on the gene expression changes induced by exoASO-STAT6 in human M2 macrophages (Fig. 2D and table S5). We identified the top 40 genes that were modulated by exoASO-STAT6 treatment and analyzed the expression of this set of genes in HCC tumors from the TCGA (The Cancer Genome Atlas) database. Correlation analysis identified a subset of 10 genes from the 40-gene list that are coherently expressed across the HCC tumors (Fig. 6A), which was independent from the previously published IFN-γ–related tumor inflammation signature (TIS) associated with clinical benefit from anti–PD-1 treatment (*48*). This signature is composed of genes such as IL4R and CCL2, which are associated with STAT6 signaling and immune-suppressive cytokines such as TGF-β. It should be noted that immune-inflammatory genes such as IL1B are also part of the signature, suggesting that the macrophages in the TME may be composed of both inflammatory and immune-suppressive phenotype (*9*, *40*, *49*). The expression of this list of 10-gene STAT6 signature was compared with other cell type–specific immune cell markers (B cells, T_regs_, macrophages, and NK cells) and TIS signature in the HCC TCGA cohort. Unsupervised clustering identified three molecular subsets in this cohort: immune cell–infiltrated (inflamed), immune cell–poor/noninflamed, and macrophage-rich/CD8 T cell–excluded subsets. On the basis of the gene expression pattern, STAT6 signature–enriched tumors were present in both the immune cell–rich tumors and macrophage-rich/CD8 T cell–poor tumors (Fig. 6B). Among the immune cell–infiltrated tumors, STAT6 signature was enriched in tumors with high macrophage, B cell and T_reg_ markers, and in a subset of cases in tumors with a high TIS (Fig. 6B). The STAT6 gene list is also expressed in a subset of tumors within the CD8 excluded, coinciding with higher levels of macrophage genes (Fig. 6B). The STAT6 signature is expressed across other TCGA indications at moderate to high levels (fig. S6A). High incidence of liver metastasis is observed in most of these indications, suggesting that exoASO-STAT6–based therapy could be helpful in these patients. Next, we evaluated the impact of STAT6 signature on disease progression primarily in HCC and other STAT6 signature high indications. High expression (quartile cutoff) of the STAT6 signature correlated with poorer survival in HCC (Fig. 6C, *P* = 0.012 for HCC) and other indications (fig. S6B). When disease progression was analyzed in both the CD8-enriched and CD8-low HCC tumors, the STAT6 signature negatively affected survival in both cases (fig. S6C). Although IL-4 expression was generally low, it was also associated with poor prognosis in CD8-low patients (fig. S6D). Together, these results suggest that there is a clinical opportunity to target the STAT6 pathway in macrophages in HCC and that both patients with CD8-enriched and CD8-poor tumors could benefit from treatment with exoASO-STAT6.

**Fig. 7.**
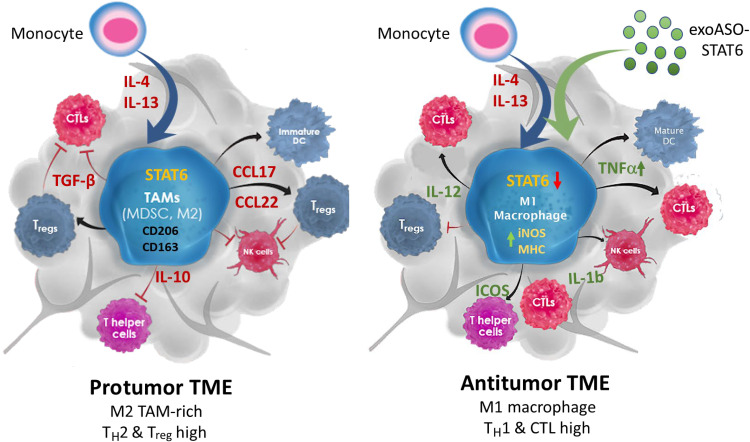
Model describing antitumor activity mediated by genetic reprogramming of TAMs by exoASO-STAT6. STAT6 expressing TAMs are critical determinants of an immunosuppressive TME by promoting recruitment of T_regs_ and inhibition of CD8 cytotoxic T cells. The ability of exoASO-STAT6 to selectively knock down STAT6 expression in immunosuppressive TAMs results in effective reprogramming to an M1 phenotype that promotes the induction of a cytotoxic immune response and an antitumoral TME. T_H_2, T helper 2; CTL, cytotoxic T lymphocytes.

On the basis of the results obtained from the CT26 and Hepa1-6 tumor models, here, we propose a model to describe the antitumor activity mediated by genetic reprogramming of TAM by exoASO-STAT6 (Fig. 7). STAT6-expressing TAMs are a determining factor in the generation of a protumoral, immunosuppressive TME by promoting recruitment of T_regs_ and inhibition of CD8 cytotoxic T cells, among other mechanisms. The ability of exoASO to knock down STAT6 in immunosuppressive TAMs results in effective reprogramming to an M1 phenotype that promotes the induction of a cytotoxic immune response and an antitumoral TME. Thus, exoASO-STAT6 treatment differs from other macrophage-targeting therapies in that it induces the replacement of dysfunctional TAMs by antitumoral TAMs, instead of causing a detrimental depletion of the entire macrophage population.

## DISCUSSION

Monoclonal antibodies inhibiting the T cell checkpoints CTLA-4 (Cytotoxic T-Lymphocyte Associated Protein 4) and PD-1 have shown antitumor immune responses, resulting in pronounced efficacy in a small percentage of patients with cancer (*50*, *51*). However, checkpoint blockade therapy is ineffective for many tumor types because of multiple immune-evasion mechanisms. One of the most common mechanisms of immune evasion is the establishment of a TME rich in macrophages with a strong immunosuppressive (M2) phenotype, which correlates with lack of responsiveness to CPI (*2*). These TAMs contribute to tumor progression by promoting tumor cell growth, survival, genetic instability, metastasis, and resistance to cytoreductive therapies, leading to poor outcomes (*5*). There have been extensive preclinical and clinical studies targeting these cells, in the hopes of reestablishing antitumor immune responses. However, these therapies, which focus primarily on inhibiting recruitment and survival of TAMs, have failed to show single-agent activity (*4*, *5*, *10*, *11*). Here, we demonstrate that treatment with exoASO-STAT6, a novel exosome-based targeted gene silencing approach to induce TAM reprogramming, results in remodeling of the TME and robust monotherapy activity in preclinical tumor models. Thus, exoASO-STAT6 is a promising new therapeutic candidate for myeloid cell targeting in cancer.

Targeting the key transcriptional networks that control M2-polarized states in macrophages is a novel approach to directly switch the genetic program in TAMs. The STAT6 transcription factor was chosen for these studies because it is a key regulator of the macrophage M2 transcriptional program in the TME in several human cancers (*22*). Currently, there are no specific and selective small-molecule inhibitors of STAT6. Although AS1517499 has been described as a STAT6 inhibitor, the specificity and selectivity have not been characterized (*52*). Furthermore, because of the structural similarity between the STAT family members, the feasibility of generating a small-molecule inhibitor that is selective and specific for STAT6 will be challenging (*53*). Therefore, the use of RNA-targeted therapies is an attractive modality to selectively target STAT6. In addition, because many transcription factors operate in multiple cell types, often regulating different gene networks and cellular functions, specificity of cell targeting is also a key requirement for the development of an ideal therapeutic candidate. In addition to its role in TAMs, STAT6 plays an essential role in CD4 T helper 2 responses (*54*), while in B cells, it promotes immunoglobulin (Ig) class switching to IgE and IgG1, as well as antigen presentation (*32*). When dosing systemically, selective targeting of STAT6 in TAMs and myeloid cells is therefore essential for an optimal therapeutic response. In this study, we demonstrate that exoASO-STAT6 combines the specificity of antisense technology and the preferential TAM tropism of our engineered exosomes to achieve this goal.

Despite significant advancements made in the field of ASOs, cell-selective delivery remains a challenge to overcome dose-limiting toxicities shown by several ASO drugs (*55*–*57*). Currently, there is no delivery methodology to enhance ASO delivery to TAMs. We have previously shown that exosomes improve the pharmacological properties of various drug modalities like STING (Stimulator Of Interferon Response CGAMP Interactor 1) agonists and cytokines (*13*, *21*). In this study, we demonstrate that exosomes enable preferential targeting of immune-suppressive TAMs and other myeloid cells in vivo, with minimal targeting of lymphocytes and nonimmune cells. While some uptake was observed in M1 macrophages in vitro, it is important to note that, in these cells, STAT6 is not phosphorylated and therefore not active (fig. S1I). This exosome-mediated preferential tropism resulted in enhanced STAT6 silencing in macrophages and myeloid cells as compared to untargeted free ASO both in vitro and in vivo. Enhanced STAT6 silencing by exoASO-STAT6 resulted in potent reprogramming of macrophages in vitro. exoASO-STAT6 was capable of reprogramming macrophages in the presence of a cocktail of immunosuppressive cytokines (TGF-β, IL-10, and IL-4) commonly found in the TME. The functional reprogramming was confirmed by a series of gene expression analysis and cytokine induction studies in M2-polarized macrophages. Treatment with exoASO-STAT6 resulted in a substantial loss of M2 macrophage phenotype. Concomitantly, robust induction of M1 gene signature and proinflammatory cytokines was observed with exoASO-STAT6 treatment. The degree of reprogramming was superior using exoASO-STAT6 as compared to free ASO, reflecting the increase in ASO potency conferred by exosome delivery (Fig. 2). In vivo, the difference between exoASO-STAT6 and free ASO is enhanced, as observed by the substantial tumor growth inhibition induced by exoASO-STAT6 compared to the lack of activity of free ASO at the same dose. This contrast reflects the ability of exosomes to more efficiently deliver ASOs to macrophage/monocyte populations in vivo (Fig. 1E) and highlights the advantage and differentiation of the exosome delivery platform. This study provides the first demonstration for the use of exosomes to deliver ASOs to the desired target cell type, improve STAT6 knockdown in TAMs, and reprogram the macrophages in the presence of immune-suppressive cytokines. Moreover, the advantages of exosome-mediated delivery of ASO are reflected in the results from our in vivo studies, which show that the effective antitumoral dose of exoASO-STAT6 is 50 to 100 times lower compared to preclinical studies previously reported with untargeted ASO therapies (*58*). Exosomes provide a novel, nonimmunogenic way of delivering complex macromolecules and provide clear advantages over other targeted or nontargeted nanoparticle-based delivery systems by reducing toxicity and potentially widening the therapeutic window (*18*, *21*, *59*–*61*). Although we have not performed head-to-head comparisons with other delivery modalities in this work, we have previously demonstrated that exosomes are nonimmunogenic and preserve viability of macrophages as compared to lipid nanoparticle formulations (*21*).

In vivo studies with exoASO-STAT6 in two syngeneic tumor models (CT26 and Hepa1-6) consistently demonstrated potent single-agent activity. Following intratumoral administration, monotherapy with exoASO-STAT6 resulted in 60% CRs in CT26 tumors. Notably, free STAT6 ASO showed no antitumor activity at the same dose, highlighting again the enhancement in ASO therapeutic efficacy conferred by exosomes. Intravenous administration of exoASO-STAT6 in mice bearing Hepa1-6 orthotopic HCC tumors also resulted in profound reduction (62%) of tumor burden. In contrast, anti-CSF1R or anti–PD-1 therapy did not result in any measurable effects on tumor growth in either model. These results represent the first demonstration of a macrophage-reprogramming therapy with significant and robust monotherapy activity across multiple anti–PD-1 refractory tumor models. Other macrophage-targeting strategies have shown modest single-agent activity (*11*, *58*, *62*, *63*). Therapies inducing TAM depletion or inhibition of TAM recruitment through inhibition of the CSF1/CSF1R pathway have shown limited single-agent activity both in preclinical models and in the clinical setting and rely on combination strategies with CPI (*4*, *5*). In addition, blockade of monocyte recruitment using CCL2/CCR2 inhibitors prevents MDSC accumulation in the tumor but has shown limited single-agent activity and requires combination with chemotherapeutic agents (*10*). Similarly, modulation of TAM function via TREM2 inhibition (*11*) or small-molecule inhibitors of PI3Kγ also relies on combinations with CPI to demonstrate tumor growth regression in preclinical models (*11*). The potent monotherapy antitumor activity is therefore a unique attribute of exoASO-STAT6 that distinguishes it from other macrophage-targeting therapies.

In both CT26 and Hepa1-6 tumor models, monotherapy activity of exoASO-STAT6 is accompanied by a profound remodeling of the TME. One of the hallmarks of effective M1 macrophage reprogramming is Nos2 induction. *Nos2* encodes the iNOS, a key marker associated with macrophage reprogramming and antitumor immune response (*13*, *40*, *41*). Despite significant differences between CT26 and Hepa1-6 models and different routes of administration used (intratumoral versus intravenous), both models demonstrated an increase in iNOS expression that was accompanied by a reduction in immunosuppressive markers, demonstrating remodeling of the TME to a proinflammatory M1-like phenotype induced by exoASO-STAT6 therapy. scRNA-seq analysis in CT26 tumors demonstrated significant expansion of M1-like macrophage/monocyte populations (clusters c3 and c5) with increased *Nos2* expression in response to exoASO-STAT6. Similarly, histology studies demonstrated a significant induction of Nos2-positive cells in Hepa1-6 tumors following exoASO-STAT6 treatment. Previous studies have shown that iNOS expression is required for T cell recruitment by TAMs after low-radiation therapy (*41*), while anti–PD-1/CTLA-4 therapy induces a marked increase of intratumoral iNOS^+^ macrophages, coinciding with the arrival in the tumor of neoantigen-specific T cells (*40*). Similarly, the increase in iNOS expression induced by exoASO-STAT6 also marks the generation of an antitumoral immune response.

The macrophage reprogramming was accompanied by induction of CD8 T cell–dependent adaptive antitumor immune response in both tumor models. In the Hepa1-6 model, several genes associated with an antitumoral cytotoxic T cell response were up-regulated in the exoASO-STAT6 group. Histological analysis confirmed a reduction in T_regs_ and an increase in CD8 T cell infiltration. In the CT26 model, exoASO-STAT6 treatment induced activation of tumor-infiltrating CD8 T cells, and the antitumor activity of exoASO-STAT6 was abrogated by CD8 T cell depletion. Consistent with these observations, complete responders following exoASO-STAT6 therapy were resistant to secondary tumor cell rechallenge. Together, these data demonstrate that exoASO-STAT6–mediated macrophage reprogramming leads to establishment of an adaptive antitumor immune response, enabling tumor elimination.

The translational significance of our preclinical findings is highlighted by the results of the analysis of the TCGA database, which suggest that exoASO-STAT6 therapy may be particularly promising in HCC and other indications. A STAT6 gene signature was developed, composed of genes such as IL4R and CCL2, which are associated with STAT6 signaling and immune-suppressive cytokines such as TGF-β. In HCC, RCC (Renal Cell Carcinoma), sarcoma, bladder, ovarian, and stomach tumors, this signature inversely correlated with overall survival and was associated with poor treatment outcome, and therefore, it identifies a high unmet–medical need population. The STAT6 signature is associated with tumors enriched for T_regs_, B cells, macrophages, and NK cells and is associated with poor prognosis. Therefore, the STAT6 signature may represent an independent prognostic factor for poor outcomes, and this subgroup of patients may uniquely benefit from exoASO-STAT6 therapy. We anticipate that primary liver cancer or liver metastasis of other tumor types would be ideal indications to test this new therapeutic approach in the clinic on the basis of (i) exosome biodistribution showing that 95% of the injected dose accumulates in the liver, (ii) enhanced *Stat6* knockdown in liver tissue associated with intravenous exosome-mediated delivery of ASO, (iii) significant antitumoral efficacy of exoASO-STAT6 in the HCC model, and (iv) poor survival in human patients with HCC with high STAT6 transcriptional signature.

In summary, this study constitutes the first description of an engineered exosome therapeutic candidate delivering an ASO targeting a transcription factor that controls macrophage phenotype (exoASO-STAT6). Exosome-mediated delivery results in a substantial enhancement of the ASO potency to suppress STAT6 expression in TAMs and induce effective reprogramming of TAMs to an M1 phenotype. As a consequence, exoASO-STAT6 treatment triggers effective remodeling of the TME to a proinflammatory, antitumoral status and the generation of a CD8 T cell–mediated response. exoASO-STAT6 potency and specificity result in potent single-agent antitumor activity, which distinguishes this novel therapeutic approach from other macrophage-targeting therapies and highlights its clinical potential. Collectively, exoASO-STAT6 represents a first-in-class therapy to target transcription factors in TAMs in a highly selective manner with minimal systemic toxicity.

## MATERIALS AND METHODS

### Cell lines and culture

CT26.WT [American Type Culture Collection (ATCC) CRL-2638], A549 (ATCC CCL-185), and Hepa1-6 (ATCC CRL-1830) cells were purchased from ATCC. CT26.WT cells were cultured in RPMI medium (Gibco) supplemented with 10% fetal bovine serum (FBS) and 1% penicillin/streptomycin. Hepa1-6 and A549 cells were cultured in 10% Dulbecco’s modified Eagle’s medium (Gibco) with 10% FBS and 1% penicillin/streptomycin. All cell lines tested negative for mycoplasma. Polymerase chain reaction tests for detection of 21 pathogens (Impact I from IDEXX) were performed on all cell lines.

### Transfection and stable cell line selection

Protocols and procedures were established according to (*20*, *21*). In short, HEK293 cells that were adapted for suspension were grown in CDM4PERMAb medium supplemented with 4 mM l-glutamine (GE Healthcare). DNA cassettes encoding PTGFRN with and without a C-terminal green fluorescent protein tag were cloned downstream of a cytomegalovirus promoter and introduced into the HEK cells via electroporation or PEI (Polyethylenimine) (Polysciences)–mediated transfection. Stable cell line selection was achieved by adding puromycin or neomycin and routinely passaged using the cell pools returned to a viability suitable for cryopreservation (>90%). A clonal cell line expressing PTGFRN alone was selected by two rounds of limited dilution, and PTGFRN overexpression was confirmed by SDS–polyacrylamide gel electrophoresis and Western blot.

### Isolation of exosomes

Procedures and protocols for exosomes production and isolation from WT and PTGFRN^++^-overexpressed HEK293 cells were established, as described by Dooley *et al.* (*20*).

### Nanoparticle tracking analysis

The concentration of exosomes was measured using the NanoSight NS3000 (Malvern Panalytical, Westborough, MA, USA). Video images were recorded for 30 s with camera level 14, and particles were analyzed with the nanoparticle tracking analysis software (version 3.2) with a detection threshold of 5. Measurements were performed in triplicate for each sample.

### Cryogenic electron microscopy

Exosome samples at a concentration of 2E12 particles/ml were transferred to a glow-discharged R2/2 Quantifoil Holey carbon film grid and incubated for 1 min. These grids were then blotted and plunge-frozen into nitrogen-cooled liquid ethane using a Vitrobot Mk IV (Thermo Fisher Scientific). The samples were then imaged at ×13,000 magnification using a Phillips CM12 electron microscope operated at 100 kEV and captured with a TVIPS 1kx1k CCD Camera.

### Antisense oligonucleotides

Cholesterol-conjugated ASOs were purchased from Axolabs GmbH, Germany. The cholesterol TEG (Triethylene glycol) moiety was conjugated to the 5′ end of the ASO with an 18-atom hexa-ethyleneglycol linker. All ASOs were lyophilized and were subsequently reconstituted in TE buffer (Thermo Fisher Scientific). ASOs contained three locked nucleic acid (LNA) modifications on either wing, with a full phosphorothioate backbone.

Sequences: STAT6 ASO-2039: **TGA**GCGAATGGACAGGT**CTT**; STAT6 ASO-2065: **GCA**AGATCCCGGATTCG**GTC**; Scramble ASO: **ACG**TGACACGTTCGGAG**AAT**. Bold indicates LNA modifications.

### Loading and quantification of ASOs on exosomes

ASOs were reconstituted in buffer TE (Thermo Fisher Scientific). WT and PTGFRN^++^ exosomes were mixed with the either STAT6 ASO-2039, STAT6 ASO-2065, or Scramble ASO in a 1:1 ratio at room temperature and incubated for 30 min. This mixture was then ultracentrifuged at 100,000*g* for 20 min at 4C, using a table-top ultracentrifugation machine (TLA120.2, Beckman Coulter) using Optima MAX XP (Beckman Coulter) to eliminate unassociated ASO. Supernatant was decanted, and the exoASO pellet was resuspended in sterile PBS and used for downstream assays.

The Quant-iT RiboGreen RNA Assay Kit (Thermo Fisher Scientific) was used for the detection and quantification of ASO loading on exosomes, according to the manufacturer’s instructions. In brief, the free ASO was diluted to a concentration of 200 nM and serially diluted thereafter in a 1:2 dilution ratio. exoASO solution was added to sample buffer in a 1:50 dilution. Ribogreen dye was diluted 1:200 in the sample buffer, and 100 μl of the RiboGreen solution was transferred to each sample, mixed, and read on a microplate reader (CLARIOstar; excitation, 488 nm; emission, 525 nm). ASO concentrations and loading were calculated on the basis of the standard curve. All treatment concentrations were based on the amount of ASO (micromolar) loaded on exosomes.

### Generation and culture of primary human monocyte–derived M1 and M2 macrophages

Human monocytes were isolated from human whole blood using 50-ml SepMate tubes (STEMCELL Technologies) and negative-selection RosetteSep Human Monocyte Enrichment Cocktail (STEMCELL Technologies) per the manufacturer’s protocols. Enriched monocytes were plated at approximately 25 to 30 million per 150-mm petri dish (Thermo Fisher Scientific) in 25 ml of RPMI medium supplemented with 10% FBS and human recombinant macrophage CSF (M-CSF; 40 ng/ml) for M2 MDMs or granulocyte M-CSF (GM-CSF) for M1 MDMs (BioLegend). Cells were incubated for 6 to 7 days at 37°C and 5% CO_2_, with the addition of 10 ml of fresh RPMI medium on day 3. At the last day, nonadherent cells and spent medium were washed and aspirated. The adherent cells were incubated with fresh 25 ml of polarizing medium [M2: RPMI/10%FBS/M-CSF with IL-4/TGF-β/IL-10 (20 ng/ml)] (M1: RPMI/10%FBS/GM-CSF with IFN-γ and LPS (20 ng/ml)] and allowed to polarize overnight. Macrophages were detached mechanically by scraping with cell scrapers using ice-cold 5 mM EDTA-PBS buffer. Cells were plated at 75,000 to 100,000 cells per well in a 96-well flat-bottom TC (Tissue culture) plate in the polarizing medium and incubated overnight in a TC incubator at 37°C and 5% CO_2_. The next day, the cells were ready for treatment.

For uptake inhibition studies in M1 and M2 MDMs, cells were first pretreated with 10 μM cytochalasin D (Thermo Fisher Scientific), poly(I) (10 μg/ml; Sigma-Aldrich), or fucoidan (500 μg/ml; Sigma-Aldrich) for 1.5 hours, after which cells were washed with PBS and treatments initiated. For labeling exosomes for uptake evaluation, exosomes were labeled with pHrodo Red NHS dye (Thermo Fisher Scientific) according to the manufacturer’s instructions. Labeled exosomes were incubated with MDMs, and fluorescent signal was used to calculate uptake in real time by IncuCyte.

### NanoString analysis

NanoString analysis was performed on human M2 MDM lysates (nCounter Human Myeloid Innate Immunty Panel), mouse CT26 tumors (nCounter Mouse Myeloid Innate Immunity Panel), and mouse Hepa1-6 tumors (nCounter Mouse PanCancer IO 360 Panel). Cell lysate (5 μl) or total RNA (50 ng) was incubated overnight at 65°C with the Reporter Code Set and Capture Probe Set according to the manufacturer’s instructions. This was then injected into the nCounter SPRINT Cartridge and analyzed by the nCounter SPRINT Profiler. Raw files were analyzed by nSolver Analysis Software 4.0, and normalized gene expression levels were obtained. Differential expression analysis was performed with Welch’s *t* test.

### LEGENDplex cytokine induction analysis

Supernatant of treated M2 MDMs were collected from each well at the end of each time point. Thirteen cytokines [IL-12p70, TNFα, IL-6, IL-4, IL-10, IL-1β, arginase, TARC, IL-1RA, IL-12p40, IL-23, IFN-γ, and IP-10 (C-X-C Motif Chemokine Ligand 10)] were analyzed using the LEGENDplex Human Macrophage/Microglia Panel (13-plex) (BioLegend), according to the manufacturer’s instruction. Briefly, eight-point standard cocktail panel and sample cell supernatant were mixed with premixed beads (smaller and larger size capture antibody beads specific to the 13 cytokines). The eight-point standard cytokine panel and sample supernatant mixtures were incubated with assay buffer in a 96-well V-bottom assay plate for 2 hours at room temperature. After washing, a biotinylated detection antibody cocktail was added to form capture bead-analyte-detection antibody sandwiches and was incubated for 1 hour at room temperature. Streptavidin-phycoerythrin (PE) was subsequently added and incubated for 30 min, which bound to the biotinylated detection antibodies, providing fluorescent signal intensities in proportion to the amount of bound cytokines. A flow cytometer, CytoFlex LX (Beckman Coulter), segregated the cytokine-specific bead populations and quantified the PE fluorescent signal. The concentration of a particular cytokine was determined using a standard curve generated in the same assay. In vivo plasma samples were analyzed using the LEGENDplex Mouse Inflammation Panel (13-plex) (BioLegend), with the same protocol.

### Animals

Six- to 8-week-old female BALB/c and C57Bl/6 mice were purchased from the Jackson Laboratory. All animals were maintained and treated at the animal care facility of Codiak Biosciences in accordance with the regulations and guidelines of the Institutional Animal Care and Use Committee (CB2017-001). For the Hepa1-6 study, 5- to 6-week-old female C57BL/6 mice were purchased from Janvier Labs (Le Genest St Isle, France). Animal housing and experimental procedures were conducted according to the French and European Regulations and the National Research Council Guide for the Care and Use of Laboratory Animals and Institutional Animal Care and Use Committee of Oncodesign (Oncomet) approved by French authorities (CNREEA agreement no. 91).

### In vivo mouse tumor models and treatment

To establish a syngeneic CT26 tumor model, 5 × 10^5^ cells were injected subcutaneously to the right flank of mice. When tumor reaches an average volume of 50 to 80 mm^3^, the mice were randomized into treatment groups according to the experimental protocol. Tumor volume (cubic millimeters) was calculated as (width)^2^ × (length) × 0.5. Intratumoral dosing was performed on the right flank tumors, and mice were injected according to the listed treatment regimens. Intraperitoneal dosing for antibody treatment was performed BIW. Intravenous dosing for exoASO/free ASO compounds were either performed as listed: as a single dose or TIW. Mice that demonstrated CR (complete tumor remission) on day 39 after study initiation were rechallenged with injection of the 5 × 10^5^ of CT26 cells into the left flank. In each rechallenge experiment, five age-matched naïve BALB/c mice were injected as controls. For establishing the mouse HCC orthotopic model, Hepa1-6 cells (1.5 × 10^6^) resuspended in Hanks’ balanced salt solution medium were injected into the spleens for C57Bl/6 mice, after which the splenectomy was performed to excise the spleen. Intravenous dosing was performed TIW from day 4, and intraperitoneal dosing was performed BIW, after cell inoculation. For intravenous biodistribution, knockdown, and efficacy studies, CT26 tumor–bearing BALB/c mice and/or naïve C57Bl/6 mice were injected intravenous into the tail vein. Antibodies used for treatment and depletion were as follows: isotype control antibody (10 mg/kg, BioLegend), anti-CD8 (10 mg/kg, BioLegend, clone 53-6.7), anti-CD4 (10 mg/kg, BioLegend, clone GK15), anti-CSF1R (10 mg/kg, BioXcell, clone AFS98), and anti–PD-1 (10 mg/kg, BioLegend, clone RMP1-14).

### Statistics and reproducibility

Data were analyzed using the GraphPad Prism software (v. 8.1.2, GraphPad Software, La Jolla, CA, USA). A *t* test was used to determine differences between the two groups, one-way analysis of variance (ANOVA) was used to determine statistical differences among multiple groups, while a two-way ANOVA was used to determine the statistical differences between multiple groups with two independent variables. For survival analyses, statistical differences between Kaplan-Meier plots were evaluated using the log-rank Mantel-Cox test. A *P* value of <0.05 was considered statistically significant.

### Data availability

All data needed to evaluate the conclusions in the paper are present in the paper and/or the Supplementary Materials. scRNA-seq datasets are available in the National Center for Biotechnology Information Gene Expression Omnibus repository (accession number GSE174068).

## Notes

View/request a protocol for this paper from *Bio-protocol*.
