# Supplementary material for: Exosome-mediated genetic reprogramming of tumor-associated macrophages by exoASO-STAT6 leads to potent monotherapy antitumor activity
Source: Sci Adv. 2022 Feb 18;8(7):eabj7002. doi: 10.1126/sciadv.abj7002 (PMC8856615; doi:10.1126/sciadv.abj7002)
Supplement: Supplementary file 1 — Supplementary Materials and Methods Figs. S1 to S6 Tables S1 to S5 [file sciadv.abj7002_sm.pdf]

Science Advances
AAAS

Supplementary Materials for

Exosome-mediated genetic reprogramming of tumor-associated macrophages by exoASO-STAT6 leads to potent monotherapy antitumor activity

Sushrut Kamerkar, Charan Leng, Olga Burenkova, Su Chul Jang, Christine McCoy, Kelvin Zhang, Kevin Dooley, Samuel Kasera, Tong Zi, Sílvia Sisó, William Dahlberg, Chang Ling Sia, Shil Patel, Karl Schmidt, Kyriakos Economides, Timothy Soos, Dalia Burzyn*, Sriram Sathyanarayanan*

*Corresponding author. Email: dalia.burzyn@codiakbio.com (D.B.); sriram.sathy@codiakbio.com (S.Sa.)

Published 18 February 2022, *Sci. Adv.* **8**, eabj7002 (2022)
DOI: 10.1126/sciadv.abj7002

This PDF file includes:

Supplementary Materials and Methods
Figs. S1 to S6
Tables S1 to S5

SUPPLEMENTARY MATERIALS AND METHODS

Isolation of exosomes:

Procedures and protocols for exosomes production and isolation from WT and PTGFRN++ overexpressed HEK293 cells were established, as described in Dooley et al *(1)*. Chemically defined media (CDM4PERMAb) was inoculated at 0.3E+06 viable cells per mL at the 10-25 L scale using WAVE bioreactors (GE Healthcare) and were maintained at 37°C and 8.0% CO_2. Cells were grown for 9 days, and the cell density and viability of the cells was measured daily on a Vi-CELL XR cell counter (Beckman Coulter). For termination of the cell culture, cells were removed by centrifugation at 6,000 × g for 15 minutes. The cell pellet was discarded, and the clarified conditioned media was filtered using Sartopore 0.8/0.45 µm MidiCaps (Sartorius), supplemented with $MgCl_2$ to a final concentration of 1 mM, and treated with 20 U/mL Benzonase (Millipore) overnight at room temperature with gentle agitation. Nuclease treated media was concentrated 10X by tangential flow filtration using a SARTOFLOW benchtop system equipped with Pellicon 2 mini 1,000 NMWL polyethersulfone (PES) ultrafiltration cassettes (Millipore). Concentrated media was then subsequently loaded into 100 mL Quick-Seal Ultra-Clear tubes and centrifuged for 60 minutes at 133,900 × g at 4°C in a 45 Ti fixed-angle rotor in an Optima XE ultracentrifuge (Beckman Coulter). Finally, the crude exosome-containing pellets were resuspended in minimal volumes of sterile PBS for further processing.

For preparing larger scale batches, the resuspended crude pellets were adjusted to a final volume of 3 mL with sterile PBS and mixed with 9 mL of 60% iodixanol solution (OptiPrep, Sigma), which resulted in a final concentration of 45% iodixanol. This was then transferred to a 38 mL UltraClear tube (Beckman Coulter). Successive layers of lower density iodixanol solutions were carefully pipetted on top of the 45% layer: 9 mL of 30%, 6 mL of 23%, 6 mL of 18%, and 3 mL of PBS. These lower density solutions were prepared by diluting OptiPrep with a homogenization buffer (250 mM sucrose, 10 mM Tris-HCl, 1 mM EDTA, pH 7.4) to achieve the final indicated iodixanol vol/vol percentages. A density gradient was achieved by centrifuging at 150,000 × g for 16 hours at 4°C in a swinging-bucket SW 32 Ti rotor (Beckman Coulter). Exosomes were isolated from the interface between the PBS and 18% iodixanol layer by careful pipetting and transferred to a clean 38 mL UltraClear tube. An additional low speed spin at 20,000 × g for 30 minutes at 4°C was used to remove any contaminating actin and actin-binding protein species. The supernatant was filtered using a 0.22 µm PVDF sterile filter, transferred to a clean 38 mL UltraClear tube, and centrifuged at 133,900 × g for 3 hours at 4°C. The final purified exosome pellet was resuspended in a minimal amount of sterile PBS, characterized, aliquoted, and frozen at -80°C for long term storage.

Radiolabeling of exosomes and PET imaging:

For zirconium (Zr) radiolabeling of PTGFRN++ exosomes, immediately prior to use, 1M HEPES buffer, pH 7.3 was mixed with [^{89}Zr]-oxalate (3D Imaging, Little Rock, AR) to prepare a 1:10 (v:v) labeling stock solution. The DFO conjugated exosomes were then added the [^{89}Zr]-oxalate labeling stock solution, and this labeling reaction mixture was then was incubated at 37°C for 60 minutes. Following incubation, the labeling reaction mixture was purified using Zeba™ Spin Desalting Columns, 7K MWCO purification columns (ThermoFisher Scientific, MA) with 1X phosphate buffered saline (PBS) as the eluent for purification. Radioactive fractions (500 µL) were collected, analyzed via SEC-HPLC (Column: TSK gel 3000 – 30 cm x 7.8, 5 µm; Buffer: 1X PBS;

Rate: 1 mL/min), and all positive [^{89}Zr]DFO-labeled EV fractions were pooled to obtain the final product for injection.

For pharmacokinetic assessment in mouse, a single bolus intravenous injection (tail vein, IV) of [^{89}Zr]-DFO-exosomes (100mCi/ <200ml) was administered under anesthesia using a 26G needle attached to a 1ml syringe and the mice then placed into the bed of a micro-PET camera. In this instance, a dynamic 60-minute acquisition was acquired followed by a short CT scan to allow for co-registration of PET and CT images. PET data acquired was histogrammed using by Fourier rebinning and the PET images were then reconstructed using an ordered subset expectation maximum algorithm (OSEM 2D) with corrections for detector normalization, deadtime, randoms, and attenuation based on the CT scan to provide the images.

Gene expression analysis by RT-qPCR:

For in vitro experiments and analysis, cells were lysed with Cells to Ct 1-step Taqman Kit (Thermo Fisher) according to manufacturer's instructions. mRNA expression was measured using TaqMan Fast Virus 1-step master mix (Thermo Fisher) and QuantStudio™ 3 & 5 Real-Time PCR System (Thermo Fisher Scientific). Ct values for all target genes were normalized to Ct values of the housekeeping gene, GAPDH. For in vivo liver, spleen and tumor samples, tissues were collected in RNA later (Thermo Fisher) solution and stored overnight at 4ºC. The tissues were placed in 1 mL of QIAzol lysis reagent and homogenized mechanically with a bead rupture. Total RNAs were isolated using RNeasy Lipid Tissue Mini Kit (ThermoFisher Scientific) following manufacturer's instructions and quantified using NanoDrop (ThermoFisher Scientific). 500 ng of total RNAs were used to generate cDNA using SuperScript IV VILO Master Mix (ThermoFisher Scientific). Target genes expression levels were assessed by RT-qPCR using TaqMan Fast Virus 1-step master mix (Thermo Fisher) and QuantStudio™ 3 & 5 Real-Time PCR System (Thermo Fisher Scientific). Ct values for all target genes were normalized to Ct values of the housekeeping gene, RPS13.

Probes used (Thermo Fisher):

Human STAT6	Hs00598625_m1
Human GAPDH	Hs02786624_g1
Mouse Stat6	Mm01160477_m1
Mouse RPS13	Mm00850011_g1
Mouse Arg1	Mm00475988_m1
Human CD163	Hs00174705_m1
Human STAT1	Hs01013996_m1
Human STAT5B	Hs00560026_m1
Human STAT4	Hs01028017_m1
Human STAT5B	Hs00234181_m1
Human STAT3	Hs00374280_m1

WES (Protein Simple WES) Analysis:

Human monocytes were negatively enriched from whole blood using RosetteSep human monocyte enrichment cocktail (STEMCELL Technologies). The enriched cells were differentiated for 6 days with RPMI/10%FBS/M-CSF media for M2 MDMs or RPMI/10%FBS/GM-CSF media for M1 MDMs and then polarized overnight with 20 ng/mL of IL-4/IL-10/TGFβ in RPMI/10%FBS/M-CSF medium for M2 MDMs and RPMI/10%FBS/GM-CSF with 20 ng/mL of IFN-γ and LPS for M1 MDMs. The adherent macrophage cells were scraped, plated, and further polarized overnight in a 96-well flat bottom TC plate at 100K cells/well. The next day, the cells were treated with the listed compounds. The first experiment was a single time point of 4 days and the second experiment was dose duration of 1, 2, 3, 5, 7, and 10 days. Treated macrophages were lysed with RIPA buffer (ThermoFisher Scientific) plus 1X protease/phosphatase inhibitor (Sigma-Aldrich) at the end of each time point. Sample cell lysates were further diluted to stock concentration of 500 μg/mL in RIPA buffer for Western analysis using WES method (Protein Simple). Stock samples were mix to a ratio of 1:4 (1-part Protein Simple master mix: 4-part lysates), denatured at 95°C for 10 minutes, and then loaded at 3 μL/well into row A of the WES 12-230 kDa pre-filled plate (Protein Simple). Wells in row B were filled with 10 μL/well of SuperblockT20 blocking buffer (ThermoFisher Scientific). Primary antibodies, Stat6 #5397S, phospho-Stat6 (Tyr641) #9361S and beta-Actin #8457S (Cell Signaling) were diluted at 1:40 (Stat6), 1:30 (p-Stat6) and at 1:100 (beta-Actin) in SuperblockT20 buffer and 10 μL/well were loaded into appropriate well in row C. Wells in row D were loaded with 10 μL/well of HRP-conjugated secondary antibodies (Protein Simple). Wells in row E were filled with 15 μL/well of chemiluminescent substrate SuperSignal West Femto (ThermoFisher Scientific). Lastly, the wash buffer wells were filled with wash buffer and the plate spun down at 2000 rpm before running the plate in the WES instrument (Protein Simple). Protein expressions were quantified (Stat6 to loading control beta-Actin) and percentage protein knockdown assessed.

CD11b enrichment:

To enrich for CD11b cells from CT26 tumors, two tumors were pooled together to make one tumor sample. Tumors were dissociated with mouse Tumor Digestion Kit (Miltenyi Biotec) according to manufacturer's instructions. This solution was then centrifuged in the same digestion tubes at 500g for 1 minute, resuspended in the same digestion solution, and passed through a 70 μm cell strainer. Cells were then washed once with PBS + 2% FBS and resuspended in 500ul of robosep buffer. EasySep Mouse CD11b Positive Selection Kit II (STEMCELL Technologies) was then used to enrich for CD11b positive cells, according to manufacturer's protocol. These cells were then used for downstream analysis such as RNA isolation and flow cytometry.

Flow Cytometry:

Tissues and tumors were dissociated using mouse tumor dissociation kit and mouse liver dissociation kit using genleMACS (Miltenyi Biotec), according to manufacturer's instructions. This solution was then centrifuged in the same digestion tubes at 500g for 1 minute, resuspended in the same digestion solution, and passed through a 70 μm cell strainer, and resuspended in PBS. Cells were then stained with Live/Dead stain dye (IR885) and were then blocked with Fc block (eBioscience). Samples were then incubated with a master mix of flow antibodies, washed with

flow cytometry wash buffer. Data were acquired using the CytoFLEX LX flow cytometer (Beckman Coulter) and analyzed with the CytExpert flow cytometry software (Beckman Coulter).

Antibodies used for flow cytometry experiments:

Antibody	Fluorochrome	Clone
CD45	BUV395	30-F11
CD11b	BUV737	M1/70
CD11c	BV421	N418
I/A-I/E	BV510	M5/114.15.2
Ly6G	BV605	1A8
CD3	BV711	17A2
F4/80	BV785	BM8
CD49b	FITC	DX5
CD19	PE	1D3/CD19
Ly6C	PE-Cy7	HK1.4
CD45	BUV395	30-F11
CD11b	BUV737	M1/70
CD11c	BV421	N418
I/A-I/E	BV510	M5/114.15.2
Ly6G	BV605	1A8
F4/80	BV785	BM8
CD86	FITC	GL-1
CD115	PE	AFS98
CD206	APC	C068C2
Ly6C	APC-Fire750	HK1.4
CD8	BUV737	53-6.7
CD4	BV421	RM4-5
CD3	BV510	17A2
CD19	BV605	6D5
CD49b	BV711	HMα2
CD25	BV785	PC61
Foxp3	APC	FJK-16s

Immunohistochemistry:

For liver tissue, the right lobe was collected, fixed in 10% NBF for 24 hours, cut into slices (no more than 5mm thickness) and subsequently embedded in paraffin (Histowax, Histolab, Sweden). Paraffin embedded tumors were cut onto slides with a thickness of 5 µm. Slides were baked for 15mins at 60°C, deparaffinized in xylene, and rehydrated via graded alcohols. Hematoxylin-Eosin (H&E) staining was performed using standard methods. For tumor cell counts,

observations were made by manual Scoring (<20% tumor cells remaining with tumor cell morphology changing and more immune cells infiltration were called Partial responders (PR); A complete lack of tumor cells were called Complete Responders (CR); >20% tumor cells remaining were called Non-Responders (NR). For tumor cell analysis by Halo Imaging Analysis Platform, tumor and stromal cell classifier methods were used for analysis. The primary antibodies for CD8 (rabbit monoclonal; Abcam EPR21769 1:2000) and FoxP3 (rabbit monoclonal; Cell Signaling D6O8R 1:2000) were used and fluorescence staining was done by anti-rabbit HRP polymer (Cell Signaling-#8114), followed with Tyramide signal application kit (AKOYA: NEL741001KT and NEL744001KT). Cell nuclei were counterstained and mounted with (VECTASHIELD H-2000). Slides were scanned with Olympus VS120, image analysis with Indica lab software.
For ASO detection in macrophages, primary antibodies used are as follows: iNOS(abcam15323) 1:1000; Stat6(abcam217998) 1:2000; ASO (28D12) 1:1000; IBA1(ab178847)1:4000 with an anti-rabbit HRP polymer (Cell Signaling-#8114) and followed with Tyramide signal application kit (AKOYA: Fluorescein NEL741001KT TSA plus Cyanin3 NEL744001KT; TSA Plus Cyanine5 NEL745001KT and TSA biotin SAT700001EA). Cell nuclei were counterstained and mounted with (VECTASHIELD H-2000). Slides were scanned with Olympus VS120, image analysis with Indica lab software FISH-IF.
Major pathological responses (MPRs) are defined as tumors with less than 10% of viable tumor remaining. For semiquantitative scoring of inflammation, histopathology of liver tumors was detected by H&E examination and included multiple focal to coalescing nodules effacing the healthy hepatic parenchyma. Neoplastic nodules were composed of neoplastic cells with various degrees of malignant cellular features (anisocytosis, anisokaryosis, 1-4 nuclei, mitotic figures), peritumoral or/and intratumoral mononuclear inflammation and tumor core necrosis. Tumor-associated inflammation was subjectively scored by a pathologist from 0 to 3 according to the following criteria: 0, absence of inflammatory cells associated with neoplastic nodules; 1 (mild), peritumoral inflammation with occasional intratumoral inflammatory cells in less than 50% of neoplastic nodules; 2 (moderate), frequent but low to high numbers of peritumoral and intratumoral inflammatory cells mixed with neoplastic cells in most neoplastic nodules; 3 (severe) neoplastic nodules are composed of large numbers of inflammatory cells effacing and replacing neoplastic cells in most neoplastic nodules across the whole liver section.

Single Cell RNA Sequencing:

Tumors were dissociated using mouse tumor dissociation kit (Miltenyi Biotec) using genleMACS (Miltenyi Biotec), according to manufacturer’s instructions. This solution was then centrifuged in the same digestion tubes at 500g for 1 minute, resuspended in the same digestion solution, and passed through a 70 µm cell strainer, and resuspended in PBS. CD45 positive cells were enriched from dissociated cells with EasySep™ Mouse CD45 Positive Selection Kit (Stem Cell Technologies), according to manufacturer’s instruction. Cell suspensions were adjusted to 1000 cells/ul and were loaded 8,300 cells into the 10X Chromium Next GEM Single Cell 3’ Reagent Kit v3.1 (10x Genomics) to target 5,000 cell recovery. All subsequent steps including GEM generation, barcoding, cDNA amplification, and 3’ gene expression library construction were performed by following the standard manufacturer’s protocols.

10x Genomics Cell Ranger pipeline (v.1.2) was utilized to perform de-multiplexing raw base call (BCL) files, alignment (mapped to the 10X reference for mm10), filtering, barcode counting, and UMI counting with the default parameters (58). In addition, the cellranger aggr pipeline was employed to combine data from multiple samples into an experiment-wide feature-barcode matrix for further data analysis. Data analysis and visualization were performed with the Seurat package (v.3.1.5) (59) in R (v.4.0.1) (60). In brief, cells in the digital count matrix were first filtered with the cutoffs that both at least 200-9,000 genes were detected and the mitochondrial read rate < 10%. Next, the remaining data matrix was normalized by the log transformation and then scaled to 10,000 transcripts per cell. Variable genes were identified with the FindVariableGenes() function with the default parameters. Principal component analysis (PCA) was performed, and the top 20 principal components (PCs) were selected for the FindNeighbors() function. Clusters were identified with the FindClusters() function with a clustering resolution set to 0.5. To visualize the data set, the Uniform Manifold Approximation and Projection (UMAP) dimensional reduction technique was utilized. Gene signatures associated with each cluster were identified using the FindAllMarkers() function with the parameters of min.pct = 0.25 and logfc.threshold = 0.25. To annotate the cell types, we utilized multiple canonical marker genes that have been previously described in the literature.

These datasets are available in the National Center for Biotechnology Information Gene Expression Omnibus (GEO) repository, accession number: GSE174068

SUPPLEMENTARY FIGURE LEGENDS

Figure S1: Characterization of WT and PTGFRN++ exoASO-STAT6, STAT6 ASO specificity, and exosomes mediated preferential delivery of ASOs to macrophages.

(A) Representative cryogenic electron microscopy image of WT exosomes. **(B)** Representative diameter size analysis (nm) of WT and PTGFRN++ exosomes loaded with STAT6 ASO-2039 and ASO-2065, and unloaded WT and PTGFRN++ exosomes, as measured by Nanoparticle Tracking Analysis (NTA). **(C)** Immunoblots for characterization of WT and PTGFRN++ exosomes: Total protein (PTGFRN ~120kDa), ALIX, Syntenin-1, TSG101, CD9, CD63 and CD81. **(D)** Normalized gene expression analysis by RT-qPCR of STAT6, STAT1, STAT3, STAT4, STAT5A and STAT5B in A549 cells transfected with 50 nM of STAT6 ASO-2065, STAT6 ASO-2039 or Scramble ASO. **(E)** Intra-tumoral biodistribution of Cy5 labelled exoASO STAT6-2039 (WT) in the indicated immune cell populations. 1 hr post single IT dose (4μg) of exoASO STAT6-2039 (Cy5), mean Fluorescent Intensity (MFI) of Cy5 was measured and plotted as histograms from the tumors of Balb/c mice bearing subcutaneous CT26 tumors. **(F)** Reduction in protein expression of STAT6 as measured by WES in the livers of mice from **(Fig. 1H). (G)** Differential uptake of pHrodo dye labeled exoASO-STAT6-2039 (PTGFRN++) in primary human monocyte derived M1 vs M2 macrophages (MDMs), over time, as measured by quantifying the Average Object Integrated Intensity by Incucyte ZOOM software. **(H)** Changes in uptake kinetics of pHrodo dye labeled exoASO-STAT6-2039 (PTGFRN++) over time in M2 and M1 MDMs, untreated, or pre-treated with either 10μM Cytochalasin D, 10μg/mL Poly (I), or 500μg/mL Fucoidan, as measured by quantifying the Average Object Integrated Intensity by Incucyte ZOOM software. **(I)** Protein expression of total STAT6 and phosphorylated STAT6 as measured by WES, in M2 and M1 MDMs. Data are mean ± s.d. **(B, D, G, H, I)** and ± s.e.m. **(F).** *$P<0.05$, ***$P<0.001$, ****$P<0.0001$, ns: not significant. Two-way ANOVA with Sidak's multiple comparisons test **(D),** One-way ANOVA with Sidak's multiple comparisons test **(F).**

Figure S2: exoASO-STAT6-2039 and 2065 induce equal reduction of STAT6 expression that leads to reprogramming of M2 macrophages.

(A) Normalized gene expression analysis by RT-qPCR of similarity of STAT6 mRNA knockdown efficiency in M2 MDMs treated for 48h with either exoASO STAT6-2039 (WT), exoASO STAT6-2039 (PTGFRN++), or exoASO Scramble. **(B)** Normalized gene expression analysis by RT-qPCR of changes in STAT6 mRNA expression in M2 MDMs treated over the course of 7 days with exoASO STAT6-2065 (PTGFRN++), Free STAT6 ASO-2065, or exoASO Scramble. Each line represents a separate dose, and STAT6 mRNA expression was analyzed at 1, 2-, 3-, 5- and 7-days post treatment. **(C)** Reduction in protein expression of STAT6 as measured by WES, in M2 MDMs treated over the course of 14 days, treated with 2.5μM of either exoASO STAT6-2065 (PTGFRN++), Free STAT6 ASO-2065, or exoASO Scramble, normalized to housekeeping gene Beta Actin. **(D)** Heap maps of modulation of genes from M1 and M2 signature analysis from Martinez et al *(31)*, from Nanostring data from (**Fig. 2D**). **(E)** Expression and modulation of STAT3, STAT1, STAT4, STAT5a and STAT5b from nanostring gene expression analysis from **(Fig. 2D). (F)** Cytokine analysis depicting modulation of IL12p40 using a multiplex flow cytometry assay, of M2 MDMs treated for 48h (24h with LPS) with either exoASO STAT6-2039 (WT), Free STAT6 ASO-2039, or exoASO Scramble. One representative donor of four is shown. **(G)** Cytokine analysis depicting fold change modulation of

compounds vs untreated control, of IL12p40, TNFα, CCL17, IL23 and IL1β using a multiplex flow cytometry assay, in M2 MDMs treated for 48h (24h with LPS) with 2.5µM of either exoASO STAT6-2039 (WT), Free STAT6 ASO-2039, or exoASO Scramble. Fold change is calculated across 4 donors. **(H)** Normalized gene expression analysis by RT-qPCR of changes in STAT6 and CD163 mRNA expression in M2 MDMs treated for 48h with either exoASO STAT6-2065 (WT), exoASO STAT6-2039 (WT), or exoASO Scramble. One representative donor of 2 graphically presented. IC50 values across 2 donors for STAT6 and CD163 shown. **(I)** Cytokine analysis depicting fold change modulation of compounds vs untreated control, of IL12p40, TNFα, IL23 and IL1β using a multiplex flow cytometry assay, in M2 MDMs treated for 48h (24h with LPS) with 2.5 µM of either exoASO STAT6-2039 (WT), exoASO STAT6-2065 (WT), or exoASO Scramble. Fold change is calculated across 3 donors. Data are mean ± s.d. *P<0.05, ***P<0.001, ****P<0.0001. One-way ANOVA with Tukey's multiple comparisons test **(E, F)**.

Figure S3: exoASO-STAT6 treatment results in a potent monotherapy anti-tumor response in CT26.

(A) Individual mice tumor growth volumes of Balb/C mice bearing subcutaneous CT26 tumors, injected intratumorally (TIW) with PBS, exoASO Scramble (4 µg), Free STAT6 ASO-2039 (4 µg), exoASO STAT6-2039 (WT) (4µg), intra peritoneally (BIW) with anti-PD-1 monoclonal antibody (10 mg/kg) and anti-CSF1R (15 mg/kg), and a combination of exoASO STAT6-2039 (WT) (4 µg) or Free STAT6 ASO-2039 (4µg) with anti-PD-1 monoclonal antibody (10 mg/kg), n=10 mice per group, data from **(Fig. 3A). (B)** Tumor growth volumes of Balb/C mice bearing subcutaneous CT26 tumors, injected intratumorally or intravenously (TIW) with PBS, exoASO Scramble (12 µg), and exoASO STAT6-2039 (PTGFRN++) (12 µg), n=9 mice per group. **(C)** Tumor growth rates from data in **(B)** and flow cytometry analysis of the comparison of % Cy5 positive (exoASO administered only) TAMS within CT26 tumors, 1 hour post single IT (4 µg) or IV (8 µg) dose of Cy5 labelled exoASO STAT6-2039 (WT)**. (D)** Tumor growth volumes of Balb/C mice bearing subcutaneous CT26 tumors, injected intratumorally with PBS (TIW, 2wk), exoASO STAT6-2039 (PTGFRN++) (6µg) (BIW, 3wk) or (TIW, 2wk) or exoASO Scramble (6µg) (TIW, 2wk), n=10 mice per group. **(E)** Tumor growth rates from data in **(D). (F)** Normalized gene expression analysis by RT-qPCR of changes in *Stat6* mRNA expression over time (1 day-7days), from CD11b-enriched fractions of CT26 tumors injected once intratumorally with either exoASO Scramble or exoASO STAT6-2039 (PTGFRN++) (6µg). Each timepoint is normalized to exoASO Scramble for that respective timepoint. Only one exoASO Scramble timepoint shown for graphical purpose. **(G)** Tumor growth rates from data in **(Fig. 3D). (H)** Confirmation of CD8 and CD4 T cell depletion from **(Fig. 3F)** by flow cytometry. **(I)** Tumor growth volumes of Balb/C mice bearing subcutaneous CT26 tumors, injected intratumorally (TIW, 2 weeks) with PBS, exoASO Scramble (4µg) or 1E11 WT or PTGFRN++ exoASO STAT6-2039 (5.9 µg and 3.4 µg respectively). Data are mean ± s.e.m. *P<0.05, **P<0.01, ***P<0.001, ****P<0.0001. One-way ANOVA with Tukey's multiple comparisons test **(C, E, G, H),** Unpaired two-tailed student's t test **(C).** CR: complete response, ns: not significant.

Figure S4: Effective reprogramming of the tumor microenvironment by exoASO-STAT6 in CT26.

(A) Flow cytometry analysis from (**Fig 4A-E**) from pre and post CD11b enriched tumor fractions, showing confirmation and quantification of CD11b enrichment from tumors. **(B)** Flow cytometry analysis of % of macrophages within CD45+ cells or total live cells, % T cells within total live cells and % CD8 T cells within total live cells, from **(Fig. 4F). (C)** tSNE plots from scRNA seq data from (**Fig. 4G**), of intra tumoral cells from exoASO Scramble or exoASO STAT6 group, to identify individual immune cell populations. **(D)** Quantification of individual immune cell populations from (**C**), as depicted by proportion (percentage) of individual cell population over total number of cells. **(E)** Violin plots of the expression of *Gzmb, Lag3, Id2* and *Il7r* from the CD8 T cell populations from scRNA seq data in **(Fig. 4G).** Data are mean ± s.e.m. *P<0.05, **P<0.01, ***P<0.001. Unpaired two-tailed student's t test **(E).**

Figure S5: Potent target gene knockdown and anti-tumor response by systemic administration of exoASO-STAT6.

(A) Representative H&E sections of Hepa1-6 tumors from (**Fig. 5A-J**), showing quantification of tumor cells by Halo Imaging Analysis Platform. **(B)** H&E stained sections of livers from **(Fig 5. A-J),** showing infiltration of immune cells in the non-responder mice that were treated with exoASO STAT6-2039 (PTGFRN++). **(C)** Percentage (%) of tumor cells as calculated by Halo Imaging Analysis Platform from H&E stained sections of livers of C57Bl/6 mice bearing orthotopic Hepa1-6 tumors in the liver, injected intravenously with exoASO-Scramble (12 µg) (TIW, 2wk) or exoASO-STAT6-2039 (PTGFRN++) (12 µg) (TIW, 2wk), or anti-PD1 (10 mg/kg) (BIW), or a combination of exoASO-STAT6-2039 (PTGFRN++) (12µg) (TIW, 2wk) and anti-PD1 (10 mg/kg) (BIW) . Representative gross images of whole Hepa1-6 tumor livers of the listed groups at study end point are shown. **(D)** Representative H&E stained sections of Hepa1-6 tumors from **(C). (E)** Histopathological scoring and analysis of intra-tumoral inflammation from H&E stained sections of Hepa1-6 tumors from (**C**). **(F)** Representative images and quantification of STAT6 expression in whole tumor, from Hepa1-6 tumor bearing animals from (**Fig. 5A-J**), quantification was performed using Halo Imaging Analysis Platform. **(G)** Heat map depicting modulation of pathway scores of data in **(Fig. 5E)** as calculated by nSolver Analysis Software. **(H)** Pathway score analysis of data in **(Fig. 5E)** as calculated by nSolver Analysis Software, of Interferon Signaling. **(I)** Changes in body weight over time for all groups from Hepa1-6 tumor bearing animals from (**Fig. 5A-J**). Sham mice underwent the same inoculation procedures as experimental mice and were used as no-tumor controls. **(J)** Quantification of serum liver enzyme levels of aspartate aminotransferase (AST) and alanine aminotransferase (AST) at study end point from Hepa1-6 tumor bearing animals from **(Fig 5A-J).** TIW: 3x a week, BIW: 2x a week. Data are mean ± s.e.m. *P<0.05, **P<0.01, ***P<0.001, ****P<0.0001. One-way ANOVA with Dunnett's multiple comparisons test **(C),** One-way ANOVA with Tukey's multiple comparisons test **(F, H)**.

Figure S6: STAT6 gene signature correlates with poor disease prognosis in HCC.

(A) Box plot of the relative expression of the STAT6 gene signature across TCGA indications. **(B)** Kaplan-Meier curves of overall survival (OS) probability in bladder cancer, ovarian cancer, stomach adenocarcinoma, sarcoma and renal cell carcinoma (RCC), based on analysis of patients with high or low STAT6 macrophage gene signature. Results were generated using Kaplan-Meier

Plotter, log-rank Mantel-Cox test. **(C)** Kaplan-Meier curves of overall survival (OS) probability in HCC, based on analysis of patients with high or low STAT6 macrophage gene signature. Patient population was segregated into CD8 T cell enriched and non-enriched analysis based on cellular content. Results were generated using Kaplan-Meier Plotter, log-rank Mantel-Cox test. **(D)** Kaplan-Meier curves of overall survival (OS) probability in HCC, based on analysis of patients with high or low IL4 expression. Patient population was segregated into CD8 T cell non-enriched and macrophage enriched analysis, based on cellular content. Results were generated using Kaplan-Meier Plotter, log-rank Mantel-Cox test.

Supplementary Figure 1

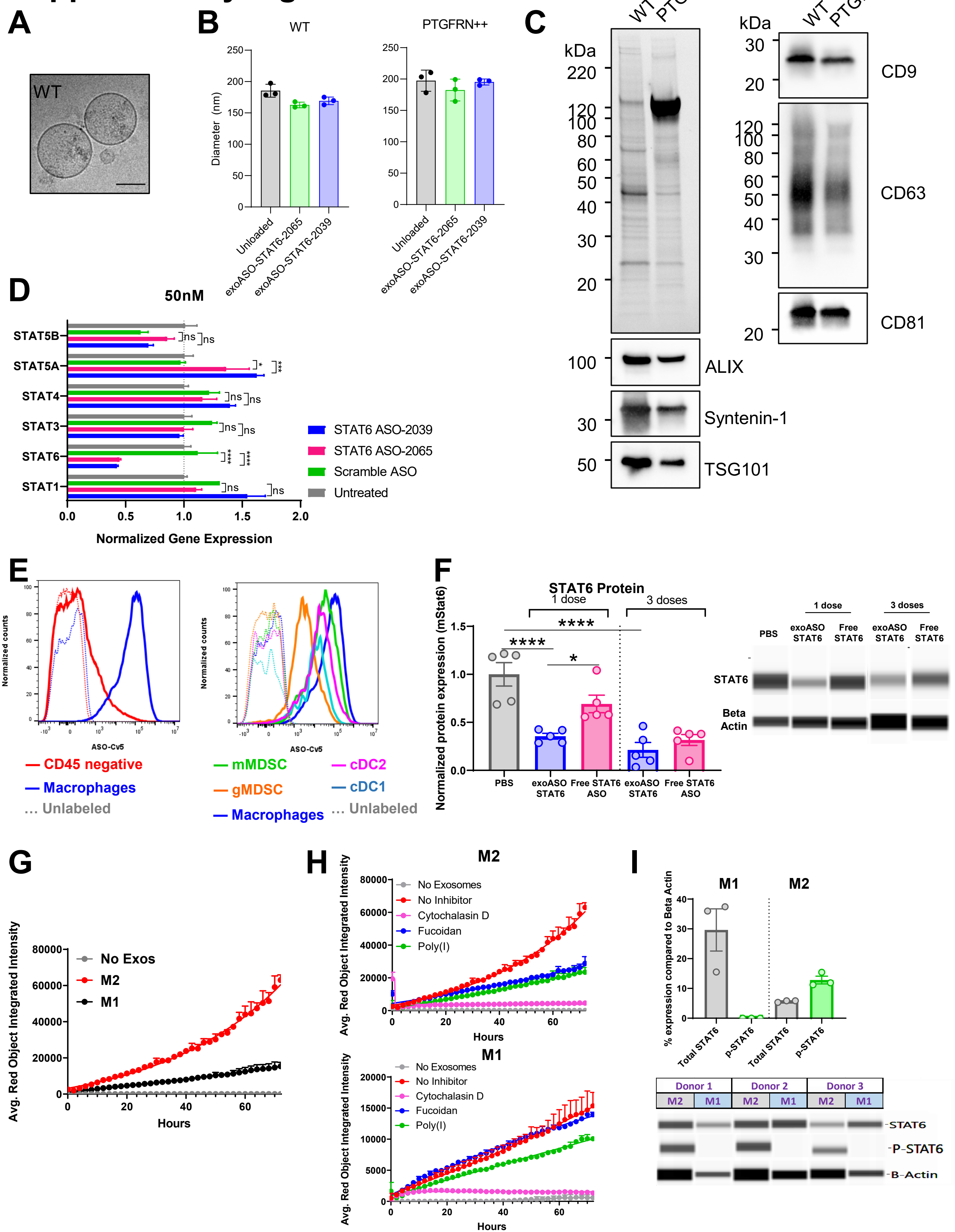

Supplementary Figure 2

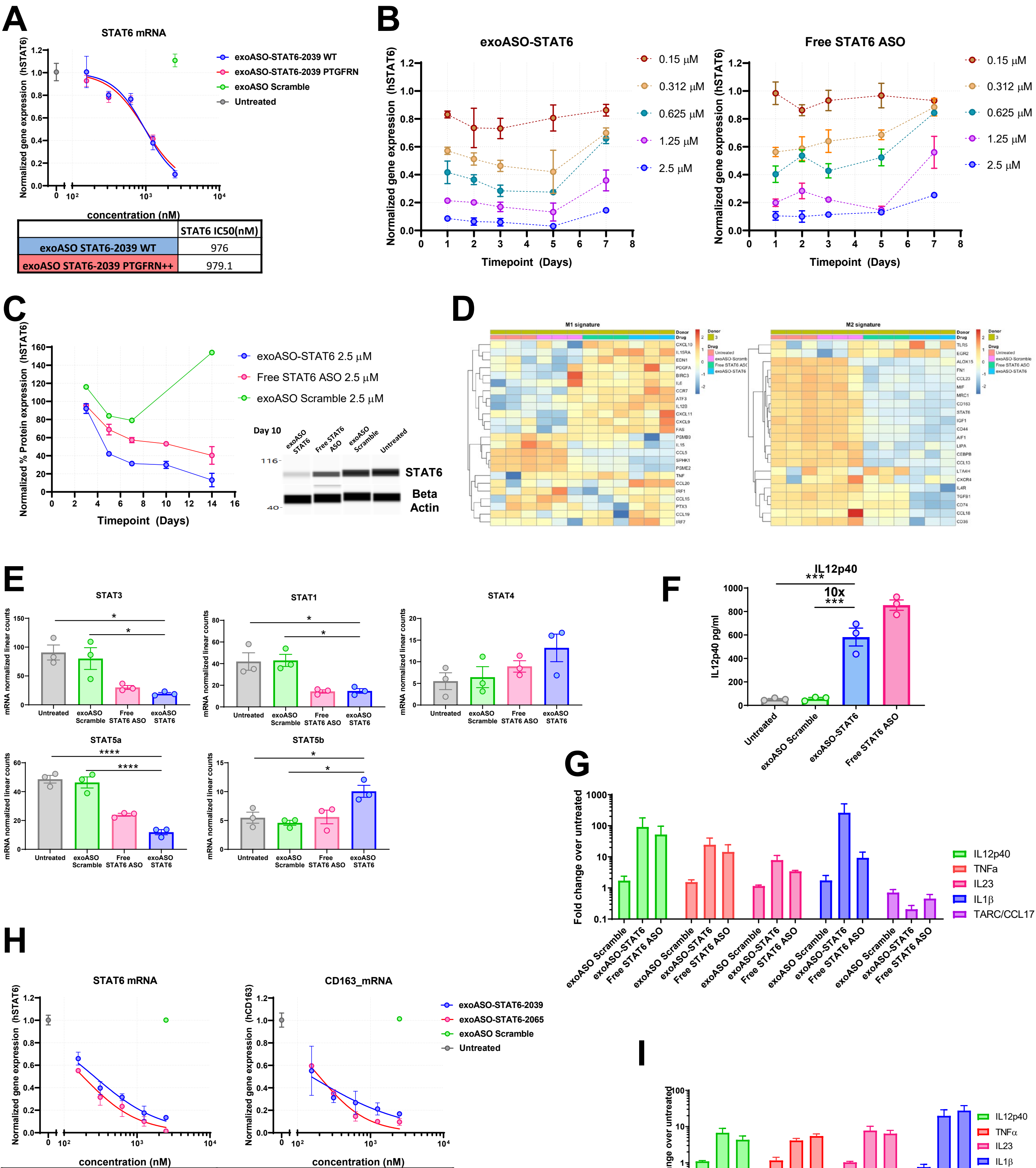

	STAT6 IC50(nM)
exoASO STAT6-2039 WT	976
exoASO STAT6-2039 PTGFRN++	979.1

	STAT6		CD163	
	D1	D2	D1	D2
exoASO-STAT6-2039 (IC50 nM)	259.4	286.8	154.1	538.1
Avg	273.1		346.1	

	STAT6		CD163	
	D1	D2	D1	D2
exoASO-STAT6-2065 (IC50 nM)	181.3	238.6	200	176.7
Avg	209.95		188.35	

Supplementary Figure 3

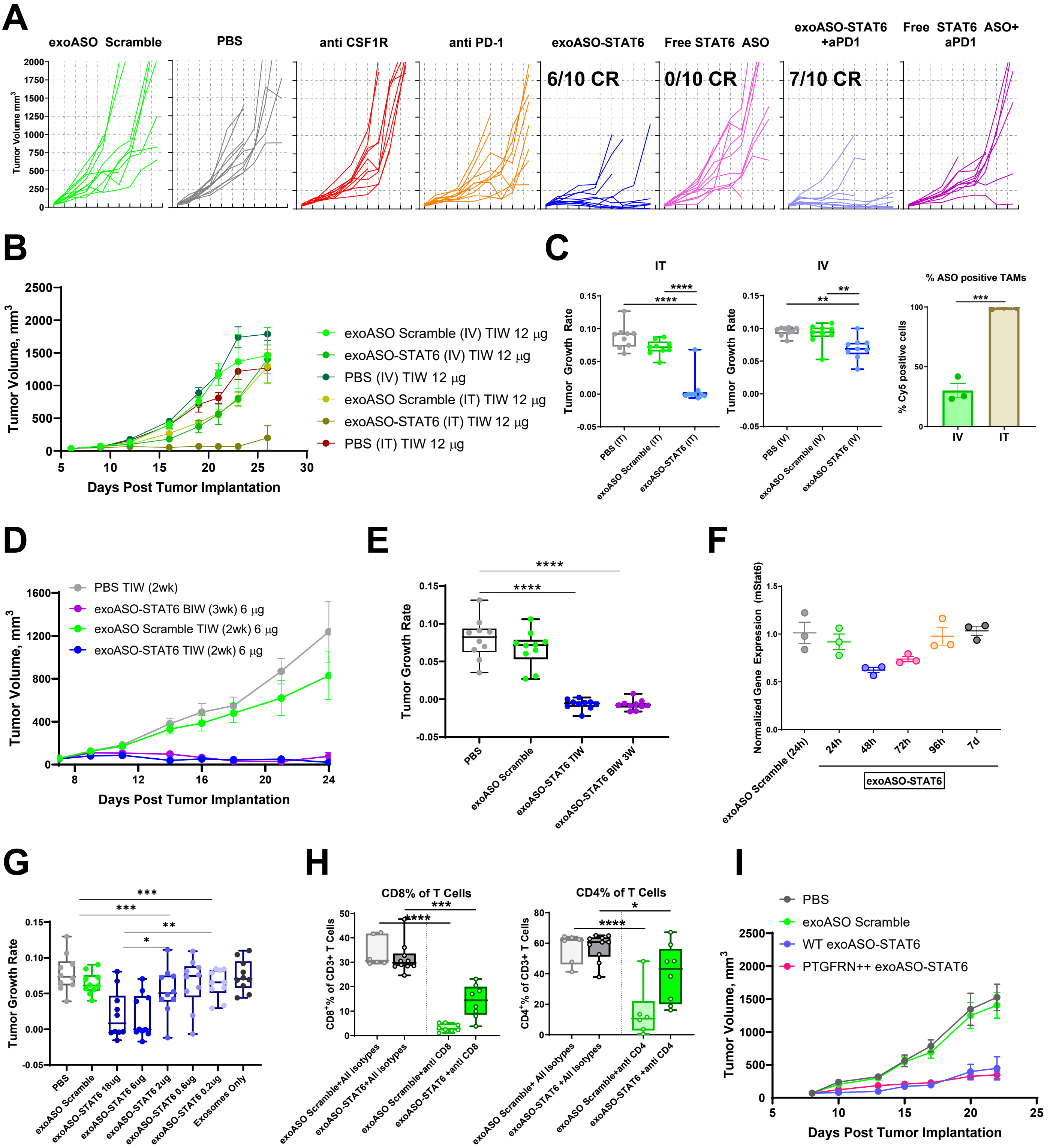

Supplementary Figure 4

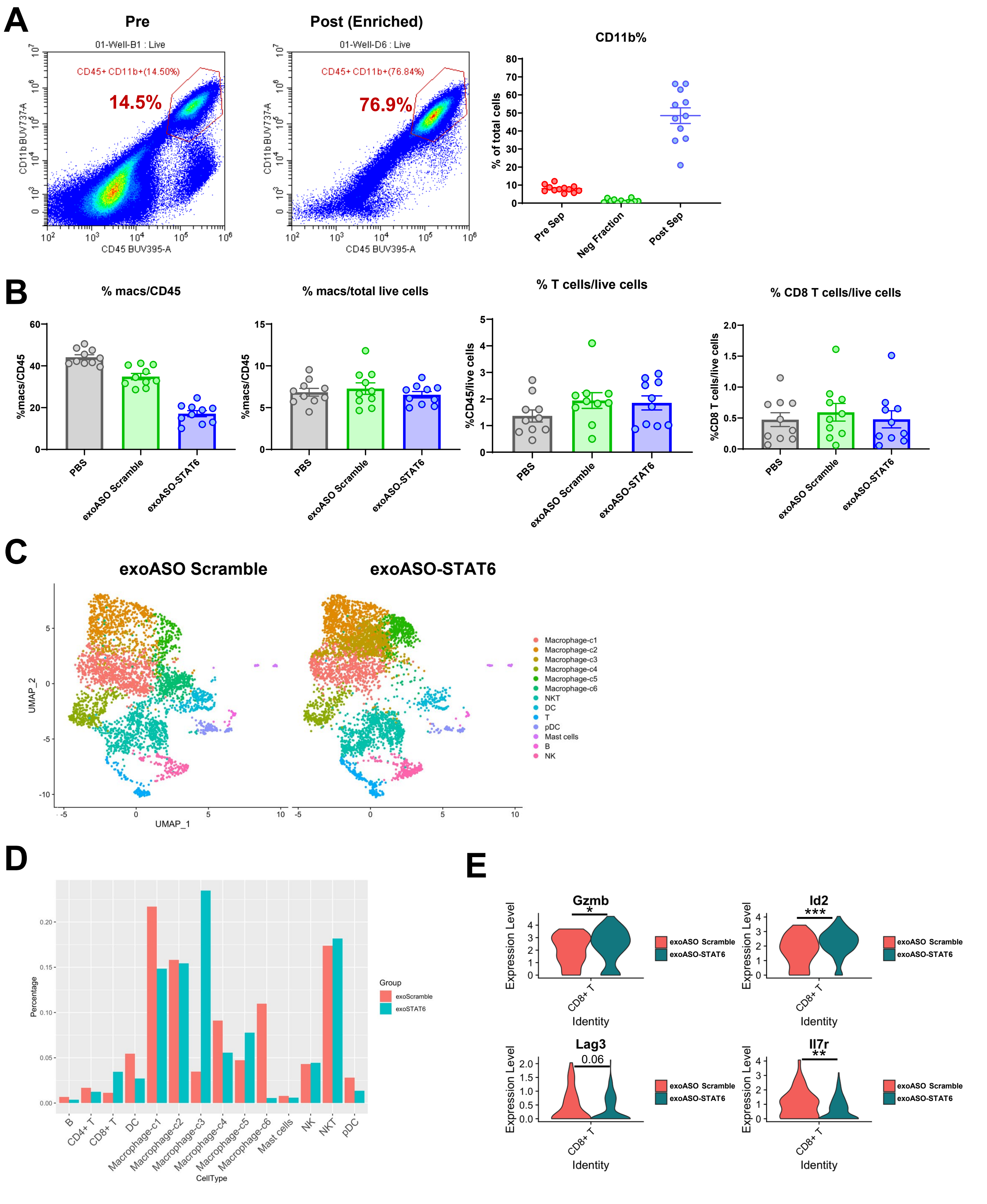

Supplementary Figure 5

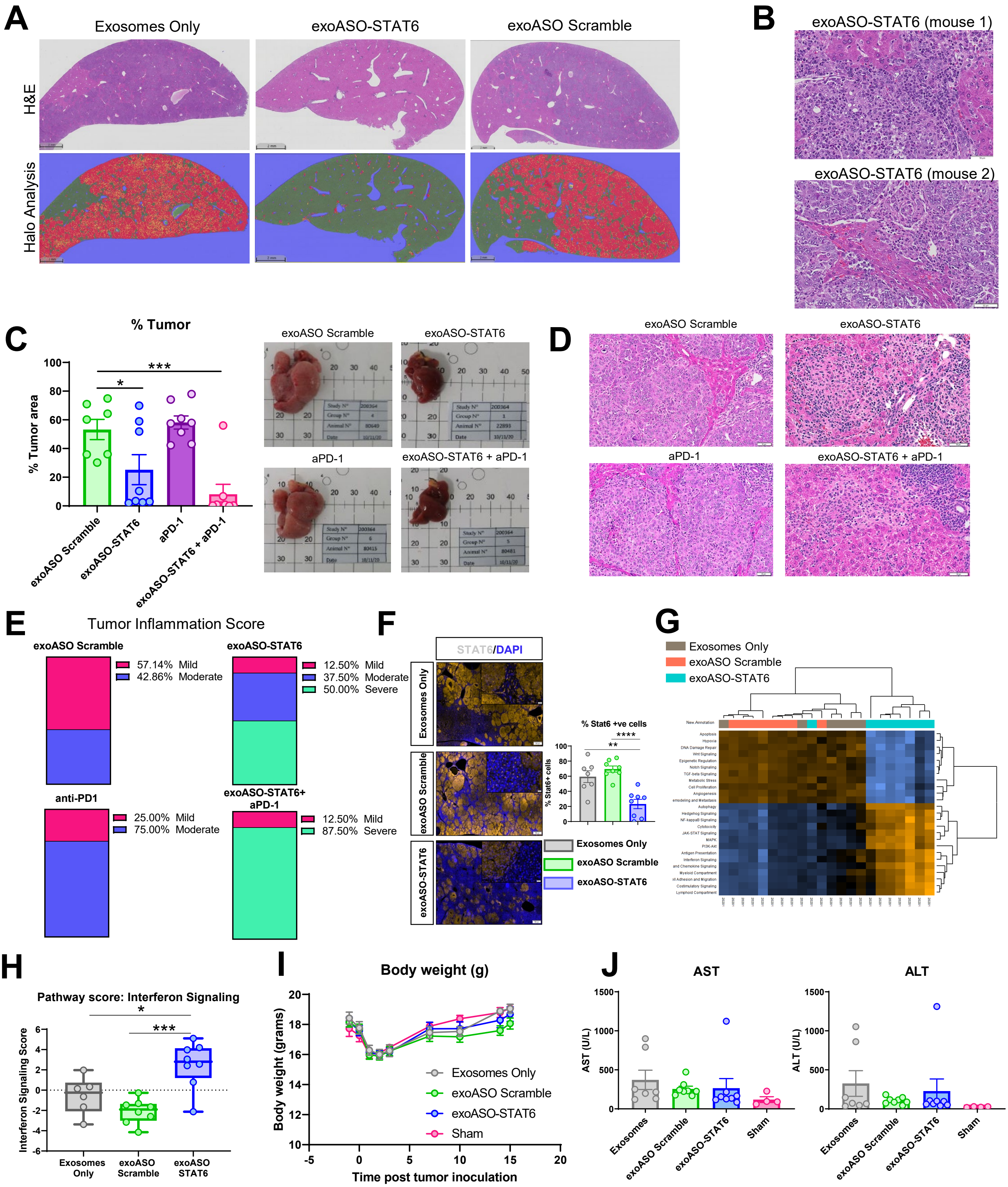

Supplementary Figure 6

A

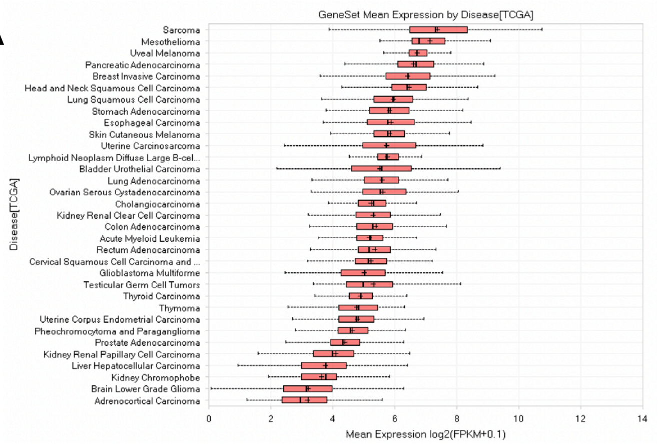

B

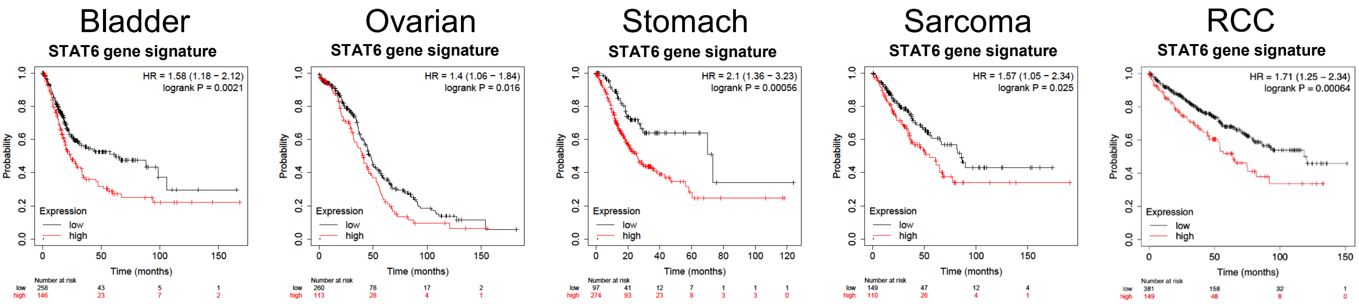

C

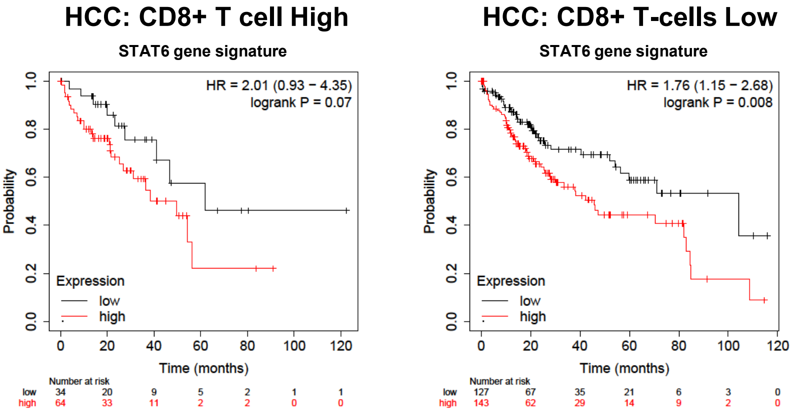

D

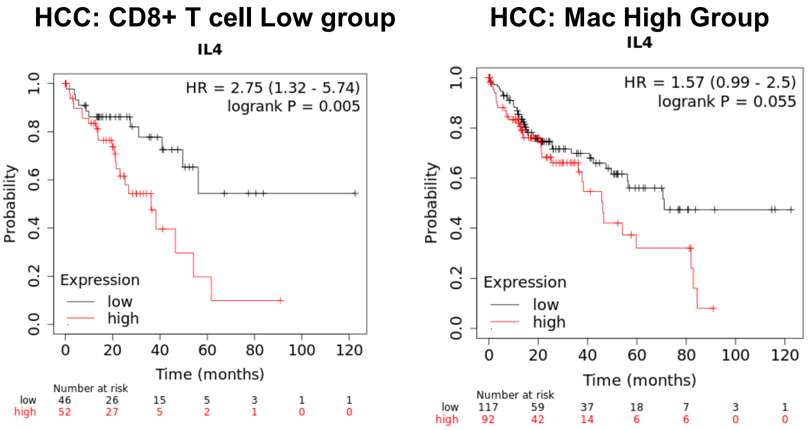

SUPPLEMENTARY TABLES

TABLE S1

	Macrophage cluster 1	Macrophage cluster 2	Macrophage cluster 3	Macrophage cluster 4	Macrophage cluster 5	Macrophage cluster 6	NK	NKT	DC	T	pDC	Mast cells	B
1	C1qa	Il1b	Rsad2	Top2a	Rgcc	Retnla	Gzma	Il2rb	Ccl22	Cd3g	Klk1	Mcpt4	Cd79a
2	C1qb	Thbs1	Marcks	Ube2c	Hilpda	Rnase2a	Gzmb	Gzmb	Ramp3	Trbc2	Ccr9	Cpa3	Ebf1
3	C1qc	Ly6c2	Ctsb	Mki67	Bnip3	Cd163	Nkg7	Prf1	Cbfa2t3	Cd3d	Siglech	Cyp11a1	Ms4a1
4	Ms4a7	Ptgs2	Sdc3	Birc5	Lgals3	Igf1	Prf1	Nkg7	Sept3	Cd3e	Cox6a2	Mcpt8	Iglc2
5	Ctsc	Cd14	Rgl1	Tubb5	Egln3	Ednrb	Ctla2a	Trbc1	Cd209a	Ctla4	Cyb561a3	Gata2	Fcmr
6	Ccnd1	Clec4e	Hmox1	Stmn1	Il7r	Ccl24	Il2rb	Gzma	Il4i1	Trac	Bcl11a	Fcer1a	Ly6d
7	Trem2	Gpr141	Psap	Pclaf	Arg1	Mrc1	Ms4a4b	Trbc2	Tbc1d4	Icos	Iglc3	Hs3st1	Pax5
8	Cd72	Plaur	Ifit2	Hmgb2	Ero1l	Cltc	Ncr1	Thy1	Timd4	Tnfrsf4	Atp1b1	Cd200r3	Pou2af1
9	Cd81	F10	Il18bp	Hist1h1b	Ndrg1	Ap2a2	Txk	Ms4a4b	Lsp1	Cd2	Klk1b27	Ms4a2	Tnfrsf13c
10	Cd63	Ccr2	Irf7	Tuba1b	Ctsl	Selenop	Klrd1	Cd3g	Syngr2	Cd8b1	Fcrla	Il13	Mzb1
11	Cadm1	Cebpb	Plin2	Cdk1	Hmox1	Maf	Serpinb6b	Satb1	Ccr7	Cxcr6	Cd7	Mrgprb1	Igkc
12	Sash1	S100a11	Clec4a1	Cdca8	Ftl1	Dazap2	Satb1	Icos	Cst3	Cd28	Pacsin1	Tph1	Iglc1
13	Camk1	Cdkn1a	Oas3	Cenpf	Plin2	Ccl6	Ctsw	Serpinb9	Cacnb3	Tnfrsf18	Mctp2	Il4	Iglc3
14	Hexa	Lst1	Slfn5	Smc2	Nos2	Ccl9	Gimap4	Klrd1	Cytip	Pdcd1	Spib	Tpsb2	Ighd
15	Grn	Mcemp1	Ifi44	Cdca3	Slc2a1	Junb	Sept1	Sh2d2a	Mmp25	Lat	Mzb1	Tpsab1	Bank1
16	Lyz2	Jarid2	Cd274	Tpx2	Clec4d	Gm42418	Trbc1	Vps37b	Gcnt2	Bcl11b	Smim5	Ctsg	Cd79b
17	Pld4	Prdx5	Grina	Prc1	Inhba	Lgmn	Car2	Cd3d	Pkib	Cd8a	Tmem108	Serpinb1a	Bcl11a
18	Cd93	Socs3	Ifi202b	Cks1b	F10	Fcgrt	Sh2d2a	Tnfrsf9	H2-Aa	Thy1	Gm21762	Cma1	Siglecg
19	Adgre1	Malt1	Oasl2	Cenpe	Basp1	Clec10a	Eomes	Ets1	H2-Eb1	Cd6	Rnase6	Hdc	Ralgps2
20	Cxcl16	Ms4a4c	Ednrb	Smc4	Fth1	Mafb	Klre1	Serpinb6b	Napsa	Lck	Sla2	Il1rl1	Cd55
21	Plau	Vcan	Ifi204	Pttg1	Cstb	Cbr2	Trdc	Cd3e	Atox1	Itk	Runx2	Klf5	Ighm
22	Ch25h	Tlr2	Gm15056	Racgap1	Card19	Marcks	Skap1	Lck	H2-DMb2	Gimap3	Ighm	Sytl3	Ccr7
23	Dab2	Plac8	Lgmn	Ccna2	P4hb	Gas6	Serpinb9	Ikzf2	Il1r2	Cd27	Sell	Hgf	Scd1
24	Fcgr3	Nfkbia	Maf	Rrm2	Adam8	F13a1	Ptprcap	Ablim1	Cd74	Ptprcap	Tubgcp5	Cish	Gm8369
25	Msr1	Csf2rb	Cfb	Ccnb2	Mmp12	Dusp1	Serpinb9b	Ctla2a	Gnb4	Skap1	Rell1	Socs2	Mef2c
26	C3ar1	Ltb4r1	Oasl1	Tk1	Srgn	Jund	Gimap3	Trac	Cd24a	Cd5	Rabgap1l	Furin	Rps27
27	Timp2	Cybb	Fgl2	Nusap1	Prdx6	Gda	Gem	Gimap3	H2-Ab1	Ms4a4b	Tcf4	Cyp4f18	BE692007
28	Slamf9	Samhd1	Ifit1	Ccnb1	Mxi1	Timp2	Bcl2	Ptprcap	Etv3	Ets1	Tex2	Il6	4930523C07Rik
29	Clec12a	Slc7a11	Ctsd	Cdc20	Grina	Klf6	Vps37b	Txk	Fscn1	Sept1	Abhd17b	Itk	Cd37
30	Hexb	Osm	Creg1	Incenp	Mmp8	Zfp36l1	Ablim1	Cd28	S100a11	Sh2d2a	St8sia4	Ier3	Serp1
31	Gatm	Nlrp3	Isg15	Hist1h2ap	Vegfa	Egr1	Ets1	Bcl2	Myo1g	Il2rb	Blnk	Slc7a5	S1pr1
32	Stab1	Nrg1	Slfn4	H2afx	Pdpn	Klf4	Klra3	Cd8a	Tuba1a	Gimap4	Mef2c	Ctse	Jund
33	Ctss	Ccrl2	Ctsl	Tmpo	Cxcl3	Mgl2	AW112010	Ctla4	Rel	Ltb	Tspan13	Cd9	Sell
34	Igfbp4	Hp	Gbp4	Ran	Slc7a2	Glul	Irf8	Nabp1	Mgl2	Ablim1	Slpi	Inpp4b	Smc6
35	Unc93b1	Sod2	Ccl8	Anp32b	Cxcl2	Ap1b1	Ptprc	Gimap4	St8sia4	Il2ra	Dnajc7	Csf1	Foxp1
36	Apoe	Ifitm6	Wfdc17	H2afz	Smox	Aplp2	Ugcg	Skap1	Crip1	Nkg7	P2ry14	Gcnt1	Napsa
37	Itm2b	Cytip	Dusp1	Ube2s	Mmp13	Ccl8	Ptpn22	Nrgn	Traf1	Tnfrsf9	Nucb2	Neat1	Gimap6
38	Snx2	Ly6i	Saa3	Nucks1	Fabp5	Trf	Klrk1	Ptpn22	Ncoa7	Trbc1	Gpr171	Csf2rb	H2-DMb2
39	Cfp	Ier3	Cmpk2	Cks2	Slpi	Stard8	Ifngr1	Klrc1	H2afz	Ptpn22	Tcf12	Tax1bp1	Zbtb20
40	Ccl12	Cxcl2	Tnfaip2	Tubb4b	Mt1	Zfp703	Selplg	Cd8b1	Map4k4	Vgll4	Irf8	Csf2rb2	Blnk
41	Pmp22	Smox	Fabp5	Slamf9	Errfi1	Klf2	Dennd4a	Ctsw	H2-DMb1	Satb1	Smc6	Gm20186	Stk17b
42	Ccl7	Vegfa	S100a9	Ranbp1	Thbs1	Fosb	Thy1	Tnfrsf18	Ifitm1	Vps37b	Bst2	Ifitm1	Ets1
43	Fcgr4	Slpi	Prdx1	C1qa	Phlda1	Idh1	Vgll4	Ncr1	Wdfy4	Gzmb	Selplg	Fosb	Satb1
44	Fcgr2b	Inhba	G0s2	C1qb	Cxcl1	Lbh	Tnfrsf9	Cxcr6	Serpinb6b	Ikzf2	Cadm1	Ctla2a	Cd69
45	Cxcl9	Cd74	S100a8	C1qc	Plk2	Rhob	Il18rap	Itgal	Tnip3	Klrd1	Xbp1	Egr3	Dnajc7
46	Mrc1	Id3	Chil3	Cenpa	Ptgs2	Wwp1	Gzmc	Ltb	Tmem123	Smad7	Mpeg1	Rgs1	Slamf6
47	Ccl2	H2-Aa	Arg1	Gatm	Hbegf	Fn1	Xcl1	Gzmc	Naaa	Prf1	Pld4	Ccl3	Rabgap1l
48	Atp1b3	Il1a	Cd36	Ch25h	Il1a	Rnase4	Cemip2	Cemip2	Serpinb9	Uhrf2	Pltp	Ccl4	Pde7a
49	Iigp1	H2-Ab1	Pf4	Pcna	Pf4	Wfdc17	Ccl5	Stat3	Samsn1	Rora	Plac8	Plk3	Fchsd2
50	Vcam1	Cxcl10	Ccl24	Ccl12	Hspa1a	Hal	Gzmd	Gzmf	Ccl5	Gzmf	Ncf1	Frmd4b	Tsc22d3

Table S1: Clusters identified by single cell RNAseq in the CT26 tumor infiltrate.

TABLE S2

	Macrophage Cluster 3					
		p_val	avg_logFC	pct.1	pct.2	p_val_adj
1	Cxcl9	2.56E-28	1.387886	0.634	0.266	5.29E-24
2	Rps12	1.44E-23	0.407943	1	0.994	2.96E-19
3	Ftl1	6.64E-23	-0.41809	1	1	1.37E-18
4	Rpl23	3.87E-22	0.372373	0.993	0.977	8.00E-18
5	Rps24	1.84E-20	0.373368	1	0.979	3.80E-16
6	Cstb	6.49E-20	-0.41189	0.959	0.988	1.34E-15
7	Rpl18a	1.81E-19	0.396118	0.979	0.951	3.74E-15
8	Igfbp4	1.84E-19	0.503659	0.324	0.085	3.80E-15
9	Hmox1	5.57E-19	-0.68678	0.766	0.952	1.15E-14
10	Rps4x	6.59E-19	0.435325	0.993	0.964	1.36E-14
11	Rpl13a	3.58E-18	0.442749	0.972	0.949	7.39E-14
12	Fth1	4.16E-18	-0.64596	1	1	8.59E-14
13	Rps20	6.04E-18	0.376904	0.993	0.987	1.25E-13
14	Apoe	6.99E-18	0.780726	0.952	0.781	1.44E-13
15	Rpl13	8.04E-18	0.38878	0.986	0.979	1.66E-13
16	Grina	1.75E-16	-0.52504	0.766	0.925	3.61E-12
17	H2-Ab1	1.88E-16	0.821725	0.876	0.78	3.87E-12
18	Rps19	1.96E-16	0.362926	0.979	0.966	4.05E-12
19	Rpl27a	2.08E-16	0.323732	1	0.987	4.30E-12
20	Atp1b3	3.93E-16	0.532755	0.89	0.892	8.11E-12
21	Cd86	4.52E-16	0.429905	0.448	0.335	9.34E-12
22	Rpl30	7.22E-16	0.347924	0.986	0.968	1.49E-11
23	Clec4d	1.07E-15	-0.70028	0.531	0.787	2.20E-11
24	Rps11	1.32E-15	0.41841	0.993	0.975	2.72E-11
25	Eef1a1	1.59E-15	0.27884	1	0.997	3.29E-11
26	Tpt1	1.85E-15	0.245561	1	1	3.81E-11
27	Trf	2.01E-15	0.581443	0.531	0.339	4.15E-11
28	Por	2.20E-15	-0.4174	0.503	0.825	4.55E-11
29	Ccnd1	2.31E-15	0.608515	0.462	0.303	4.78E-11
30	Rpl19	4.48E-15	0.299707	0.993	0.981	9.25E-11
31	Rps16	4.94E-15	0.326395	1	0.988	1.02E-10
32	Rps10	8.27E-15	0.356878	0.986	0.98	1.71E-10
33	Ell2	9.12E-15	-0.55826	0.31	0.639	1.88E-10
34	Rps15	9.54E-15	0.409783	0.986	0.965	1.97E-10
35	Rpl28	9.88E-15	0.372546	0.993	0.96	2.04E-10
36	Rps9	1.43E-14	0.337039	0.986	0.996	2.96E-10
37	Rpl6	1.77E-14	0.326065	0.993	0.96	3.65E-10
38	Rpl23a	3.35E-14	0.374767	0.966	0.933	6.92E-10
39	Rpl10a	7.38E-14	0.403634	0.966	0.922	1.52E-09
40	Lgals3	8.43E-14	-0.58529	0.945	0.981	1.74E-09
41	Rps27a	9.95E-14	0.293781	1	0.981	2.05E-09
42	Gm10076	1.18E-13	0.292342	0.545	0.505	2.44E-09
43	Glrx	1.74E-13	-0.56945	0.655	0.876	3.59E-09
44	Cd81	1.82E-13	0.410449	0.345	0.116	3.75E-09
45	Cd36	2.42E-13	-0.94507	0.193	0.449	4.99E-09
46	Rps5	2.57E-13	0.360872	0.986	0.976	5.30E-09
47	Rps7	2.64E-13	0.356396	1	0.96	5.44E-09
48	Eef1b2	3.26E-13	0.370191	0.952	0.881	6.72E-09
49	Lilr4b	7.02E-13	-0.42412	0.793	0.927	1.45E-08
50	Rpl32	1.34E-12	0.37917	0.986	0.967	2.77E-08

	Macrophage Cluster 5					
		p_val	avg_logFC	pct.1	pct.2	p_val_adj
1	Nos2	3.55E-23	-1.0948	0.333	0.73	7.33E-19
2	Rps3a1	4.91E-23	0.391922	0.995	0.975	1.01E-18
3	Rpl12	5.91E-22	0.552619	0.949	0.863	1.22E-17
4	Gm15056	1.15E-20	-1.75618	0.081	0.412	2.37E-16
5	Rpl32	1.64E-20	0.504434	0.98	0.967	3.39E-16
6	Rpl3	4.26E-20	0.451053	0.944	0.875	8.80E-16
7	Rpl13a	2.00E-19	0.480926	0.955	0.919	4.13E-15
8	Rps12	3.39E-19	0.462887	0.995	0.982	7.00E-15
9	Eef1a1	2.99E-18	0.322594	1	0.99	6.18E-14
10	Lyz2	4.09E-18	0.81	0.899	0.766	8.44E-14
11	Rps4x	4.42E-18	0.412588	0.97	0.964	9.14E-14
12	Rps20	5.11E-18	0.379528	0.995	0.975	1.05E-13
13	Rps8	5.87E-18	0.388401	0.995	0.969	1.21E-13
14	Rps19	1.52E-17	0.427067	0.98	0.947	3.14E-13
15	Socs1	1.95E-17	-0.8792	0.263	0.527	4.02E-13
16	Rpl10a	2.54E-17	0.506779	0.949	0.845	5.24E-13
17	Tapbp	5.17E-17	-0.65621	0.596	0.789	1.07E-12
18	Rpl13	5.76E-17	0.419681	0.985	0.962	1.19E-12
19	Zbp1	5.92E-17	-0.5998	0.283	0.646	1.22E-12
20	Grina	7.17E-17	-0.59042	0.798	0.941	1.48E-12
21	Rps27a	7.29E-17	0.383932	0.995	0.964	1.50E-12
22	Sod2	7.74E-17	-0.70217	0.753	0.896	1.60E-12
23	Tmsb10	1.02E-16	-0.72184	0.909	0.969	2.11E-12
24	Rps5	1.45E-16	0.432161	0.96	0.952	3.00E-12
25	Ccl6	1.88E-16	0.641943	0.924	0.718	3.88E-12
26	Mrc1	1.89E-16	0.891925	0.813	0.534	3.90E-12
27	B2m	6.25E-16	-0.38365	1	1	1.29E-11
28	Rpl5	7.23E-16	0.536326	0.909	0.753	1.49E-11
29	Rack1	7.79E-16	0.447237	0.97	0.898	1.61E-11
30	Rpl14	1.09E-15	0.464316	0.929	0.83	2.24E-11
31	Ccl9	1.27E-15	0.540685	0.909	0.69	2.62E-11
32	AW11201(	2.54E-15	-1.08653	0.46	0.702	5.25E-11
33	Cstb	4.05E-15	-0.3851	0.97	0.992	8.37E-11
34	Rps11	4.19E-15	0.397807	0.98	0.957	8.65E-11
35	Rpl18a	4.70E-15	0.399111	0.985	0.934	9.71E-11
36	Pf4	4.71E-15	0.840589	0.818	0.537	9.72E-11
37	Rpsa	5.26E-15	0.373682	0.955	0.934	1.09E-10
38	Rps7	6.14E-15	0.365886	0.98	0.939	1.27E-10
39	Glrx	7.38E-15	-0.77414	0.677	0.832	1.52E-10
40	Rps14	8.72E-15	0.327626	0.975	0.954	1.80E-10
41	Rplp1	9.84E-15	0.393351	0.985	0.99	2.03E-10
42	Cebpb	1.15E-14	0.383075	0.995	0.908	2.37E-10
43	Rps13	1.17E-14	0.324799	0.985	0.947	2.42E-10
44	Rps15	1.89E-14	0.415933	0.965	0.921	3.91E-10
45	Rps24	2.14E-14	0.343876	0.99	0.972	4.43E-10
46	Timp2	6.78E-14	0.741964	0.535	0.277	1.40E-09
47	Rps10	8.40E-14	0.347008	0.995	0.967	1.74E-09
48	Rpl17	1.74E-13	0.367938	0.975	0.944	3.59E-09
49	Prdx6	2.09E-13	-0.64006	0.747	0.911	4.32E-09
50	Il1rn	3.22E-13	-0.6375	0.783	0.931	6.65E-09

TABLE S2 (CONT.)

	Macrophage Cluster 3					
		p_val	avg_logFC	pct.1	pct.2	p_val_adj
51	Rplp2	1.46E-12	0.320473	1	0.976	3.01E-08
52	Cox7a2l	1.60E-12	0.360855	0.752	0.716	3.30E-08
53	Gas5	1.85E-12	0.321564	0.503	0.477	3.83E-08
54	Rplp1	2.59E-12	0.245388	0.993	0.997	5.35E-08
55	Rpl17	6.35E-12	0.350173	0.993	0.971	1.31E-07
56	Cd83	7.20E-12	0.572638	0.669	0.548	1.49E-07
57	Atp5g2	8.16E-12	0.334846	0.91	0.893	1.68E-07
58	Ctsl	8.24E-12	-0.64894	0.966	0.992	1.70E-07
59	Rps3	8.41E-12	0.321921	0.979	0.944	1.74E-07
60	Blvrb	1.02E-11	-0.49678	0.745	0.882	2.11E-07
61	Eif3f	1.40E-11	0.357628	0.91	0.86	2.89E-07
62	Fau	1.60E-11	0.264386	1	0.99	3.30E-07
63	Heatr5a	1.82E-11	0.192735	0.269	0.17	3.76E-07
64	Rpl37a	2.66E-11	0.329926	1	0.988	5.50E-07
65	Rpl5	2.89E-11	0.380935	0.945	0.874	5.96E-07
66	Il10ra	3.32E-11	0.365333	0.634	0.546	6.86E-07
67	Twf2	3.47E-11	0.263309	0.579	0.565	7.16E-07
68	Rps3a1	3.94E-11	0.273536	1	0.987	8.14E-07
69	Rpl36a	4.69E-11	0.335942	0.897	0.849	9.68E-07
70	H2-Eb1	5.17E-11	0.728322	0.821	0.687	1.07E-06
71	Slc48a1	5.59E-11	-0.47072	0.49	0.75	1.15E-06
72	Cd274	6.77E-11	-0.48477	0.717	0.893	1.40E-06
73	Dnase1l3	6.84E-11	0.361288	0.228	0.083	1.41E-06
74	Palld	6.95E-11	0.296046	0.366	0.22	1.44E-06
75	C1qb	7.15E-11	0.654351	0.662	0.594	1.48E-06
76	Prdx6	7.40E-11	-0.52589	0.476	0.697	1.53E-06
77	Ctsd	8.17E-11	-0.43154	0.952	0.992	1.69E-06
78	Rack1	8.66E-11	0.28781	0.972	0.97	1.79E-06
79	Rpl3	1.01E-10	0.331619	0.993	0.952	2.08E-06
80	Sod2	1.11E-10	-0.50337	0.786	0.909	2.29E-06
81	Rpl12	1.56E-10	0.324756	0.972	0.948	3.21E-06
82	Rps25	2.16E-10	0.284324	0.931	0.937	4.45E-06
83	Smpdl3a	2.28E-10	-0.47752	0.476	0.716	4.71E-06
84	H2-Aa	2.74E-10	0.742458	0.89	0.771	5.65E-06
85	Lyz2	2.83E-10	0.492306	0.972	0.915	5.85E-06
86	R3hcc1l	3.51E-10	0.103478	0.117	0.117	7.24E-06
87	Eif4e	3.56E-10	-0.37911	0.538	0.777	7.34E-06
88	Rpl7	3.92E-10	0.296474	1	0.972	8.10E-06
89	Commd4	4.58E-10	0.116971	0.379	0.419	9.47E-06
90	Gm14636	4.73E-10	-0.66941	0.228	0.486	9.77E-06
91	Ly86	4.77E-10	0.397399	0.731	0.625	9.86E-06
92	Cndp2	5.79E-10	-0.3295	0.91	0.963	1.20E-05
93	AA467197	6.27E-10	-0.64409	0.166	0.363	1.30E-05
94	Rps29	6.75E-10	0.184879	1	0.992	1.39E-05
95	Cd74	6.99E-10	0.65164	0.966	0.884	1.44E-05
96	Prdx1	7.07E-10	-0.58071	0.979	0.992	1.46E-05
97	Rpl18	7.13E-10	0.306328	0.966	0.963	1.47E-05
98	Ly6c2	7.19E-10	-0.64411	0.407	0.68	1.48E-05
99	Serinc3	7.30E-10	0.343362	0.883	0.859	1.51E-05
100	Csf1r	7.40E-10	0.34238	0.848	0.806	1.53E-05

	Macrophage Cluster 5					
		p_val	avg_logFC	pct.1	pct.2	p_val_adj
51	Irf7	5.17E-13	-0.54559	0.495	0.781	1.07E-08
52	S100a4	6.35E-13	0.575561	0.884	0.735	1.31E-08
53	Rpl27a	6.92E-13	0.305083	0.98	0.98	1.43E-08
54	Rps15a	1.14E-12	0.318098	0.975	0.977	2.35E-08
55	Rps3	1.47E-12	0.298836	0.98	0.941	3.04E-08
56	Rpl23	1.75E-12	0.323427	0.995	0.982	3.61E-08
57	Eef1b2	2.51E-12	0.468619	0.869	0.743	5.19E-08
58	AA467197	3.05E-12	-1.02935	0.182	0.438	6.30E-08
59	Npc2	3.49E-12	-0.37602	0.98	0.997	7.20E-08
60	Selenop	4.36E-12	0.636955	0.475	0.214	9.01E-08
61	Slamf7	6.97E-12	-0.61454	0.157	0.417	1.44E-07
62	Prdx5	8.66E-12	-0.50848	0.909	0.975	1.79E-07
63	Fth1	9.28E-12	-0.44774	1	1	1.92E-07
64	H2-T23	9.91E-12	-0.50225	0.444	0.669	2.05E-07
65	mt-Nd1	1.06E-11	0.539582	0.899	0.85	2.19E-07
66	Slc31a1	1.26E-11	-0.53055	0.399	0.656	2.60E-07
67	Rsad2	1.33E-11	-0.89516	0.318	0.623	2.74E-07
68	Slc31a2	1.36E-11	-0.40679	0.333	0.618	2.82E-07
69	Upp1	1.38E-11	-0.4806	0.399	0.705	2.86E-07
70	Gbp2b	1.59E-11	-0.55761	0.333	0.618	3.28E-07
71	Gbp2	1.67E-11	-0.60797	0.343	0.641	3.44E-07
72	Rpl7	1.84E-11	0.337044	0.99	0.962	3.81E-07
73	Rpl8	2.27E-11	0.253696	0.949	0.962	4.70E-07
74	Cd274	2.56E-11	-0.56469	0.677	0.835	5.28E-07
75	Fcgrt	5.45E-11	0.551011	0.323	0.145	1.12E-06
76	Rpl9	5.79E-11	0.298479	0.985	0.969	1.20E-06
77	Rpl22	6.47E-11	0.322706	0.919	0.901	1.34E-06
78	Calm3	6.69E-11	-0.4285	0.677	0.758	1.38E-06
79	Rplp0	7.43E-11	0.339448	0.975	0.936	1.53E-06
80	Cd300lf	9.30E-11	-0.60384	0.586	0.763	1.92E-06
81	Rpl27	1.03E-10	0.310947	0.975	0.952	2.12E-06
82	Rpl28	1.04E-10	0.357823	0.97	0.962	2.15E-06
83	Cd200	1.06E-10	-0.62371	0.086	0.333	2.19E-06
84	Rpl7a	1.29E-10	0.301745	0.944	0.898	2.67E-06
85	Rps25	1.42E-10	0.277815	0.939	0.919	2.93E-06
86	Hk3	1.59E-10	-0.47896	0.384	0.628	3.29E-06
87	Ly6c2	1.73E-10	-0.69741	0.187	0.466	3.57E-06
88	Rnf149	2.04E-10	-0.42902	0.389	0.611	4.20E-06
89	Slfn5	2.68E-10	-0.62098	0.359	0.595	5.53E-06
90	Rps26	3.20E-10	0.302229	0.97	0.947	6.62E-06
91	Mdm2	3.88E-10	-0.53187	0.672	0.809	8.01E-06
92	Rpl11	4.25E-10	0.295459	0.965	0.944	8.77E-06
93	Rps16	6.55E-10	0.276385	0.99	0.985	1.35E-05
94	Cfb	7.49E-10	-0.59157	0.278	0.499	1.55E-05
95	Inhba	1.04E-09	-0.71707	0.53	0.758	2.15E-05
96	Dtx2	1.14E-09	-0.36577	0.111	0.321	2.36E-05
97	Ifi44	1.15E-09	-0.67336	0.293	0.534	2.38E-05
98	Plac8	1.35E-09	-0.74649	0.505	0.738	2.79E-05
99	Gas6	1.35E-09	0.435777	0.167	0.038	2.79E-05
100	Psme2	1.46E-09	-0.47641	0.571	0.72	3.01E-05

TABLE S2 (CONT.)

	Macrophage Cluster 3								Macrophage Cluster 5					
		p_val	avg_logFC	pct.1	pct.2	p_val_adj				p_val	avg_logFC	pct.1	pct.2	p_val_adj
101	H2-DMb1	8.31E-10	0.520577	0.683	0.473	1.72E-05		101	Fcgr1	1.89E-09	-0.54998	0.601	0.789	3.91E-05
102	Oip5os1	8.51E-10	0.186252	0.434	0.444	1.76E-05		102	Ppia	2.06E-09	0.284314	0.955	0.962	4.25E-05
103	Pdlim5	1.16E-09	0.149774	0.372	0.415	2.39E-05		103	Ifi204	2.19E-09	-0.45734	0.54	0.766	4.52E-05
104	Tecr	1.18E-09	0.231068	0.469	0.353	2.44E-05		104	F10	2.34E-09	-0.49392	0.571	0.799	4.83E-05
105	Rpl27	1.23E-09	0.301668	0.986	0.966	2.54E-05		105	H2-K1	2.57E-09	-0.33027	0.985	1	5.30E-05
106	Rgl1	1.49E-09	-0.40273	0.724	0.881	3.07E-05		106	Slfn4	2.58E-09	-0.57845	0.172	0.433	5.33E-05
107	mt-Nd4	1.80E-09	0.28365	0.966	0.974	3.71E-05		107	Ifi47	2.97E-09	-0.49892	0.222	0.483	6.13E-05
108	mt-Cytb	2.01E-09	0.356525	0.979	0.987	4.16E-05		108	Cd83	3.11E-09	0.550536	0.581	0.374	6.43E-05
109	Rab4b	2.51E-09	0.212385	0.517	0.472	5.19E-05		109	Cd24a	3.74E-09	-0.45491	0.141	0.389	7.72E-05
110	Rpl34	2.62E-09	0.249859	0.986	0.964	5.41E-05		110	Igfbp4	4.26E-09	0.306683	0.182	0.046	8.80E-05
111	Oat	2.62E-09	0.104066	0.4	0.473	5.42E-05		111	Dab2	5.92E-09	0.465928	0.889	0.794	0.000122
112	Vcam1	2.96E-09	0.630449	0.352	0.138	6.12E-05		112	Slc9a9	7.25E-09	0.243812	0.136	0.013	0.00015
113	Isoc1	2.97E-09	0.219888	0.4	0.407	6.13E-05		113	Rpl36a	7.75E-09	0.343753	0.859	0.789	0.00016
114	mt-Nd1	2.99E-09	0.278939	0.966	0.976	6.16E-05		114	Snx2	7.77E-09	0.388496	0.657	0.565	0.00016
115	Jaml	3.32E-09	0.394598	0.648	0.521	6.85E-05		115	Gda	7.80E-09	0.435237	0.672	0.461	0.000161
116	Dock11	3.80E-09	0.206049	0.324	0.256	7.84E-05		116	Isg15	8.37E-09	-0.65661	0.379	0.621	0.000173
117	Sdf4	3.85E-09	0.174238	0.593	0.632	7.95E-05		117	Eef2	8.61E-09	0.314368	0.894	0.827	0.000178
118	Ptma	4.31E-09	0.227132	1	0.986	8.90E-05		118	Rtp4	9.35E-09	-0.44881	0.227	0.476	0.000193
119	Upp1	4.36E-09	-0.57258	0.324	0.555	9.00E-05		119	Rpl23a	9.58E-09	0.357193	0.939	0.86	0.000198
120	Saa3	4.68E-09	-0.89862	0.083	0.313	9.67E-05		120	Ptges	1.14E-08	-0.73361	0.091	0.267	0.000235
121	Sh3bp5	4.98E-09	-0.39675	0.331	0.573	0.000103		121	Oas3	1.18E-08	-0.36197	0.116	0.3	0.000243
122	Wfdc17	5.13E-09	-0.56827	0.869	0.94	0.000106		122	C3	1.19E-08	-0.29617	0.298	0.565	0.000246
123	Fcgr2b	5.39E-09	0.278309	0.931	0.906	0.000111		123	Rgl1	1.28E-08	-0.47245	0.672	0.819	0.000264
124	mt-Atp6	5.72E-09	0.281694	0.979	0.993	0.000118		124	Folr2	1.30E-08	0.439604	0.379	0.17	0.000268
125	Txn1	5.80E-09	-0.35411	0.903	0.962	0.00012		125	Rnase4	1.46E-08	0.369706	0.343	0.191	0.000302
126	Rpl39	5.83E-09	0.234376	1	0.965	0.00012		126	Car13	1.64E-08	-0.31685	0.227	0.42	0.000339
127	H2-DMa	6.25E-09	0.363502	0.862	0.769	0.000129		127	H3f3a	1.65E-08	-0.34294	0.955	0.977	0.000342
128	Sulf2	6.78E-09	0.184732	0.152	0.054	0.00014		128	Psmb9	1.69E-08	-0.37042	0.545	0.756	0.00035
129	Nop53	7.76E-09	0.187211	0.538	0.553	0.00016		129	Gbp4	1.76E-08	-0.58034	0.263	0.509	0.000364
130	Ednrb	7.99E-09	-0.66374	0.145	0.384	0.000165		130	Rplp2	1.84E-08	0.293448	0.98	0.954	0.00038
131	Plpp3	8.19E-09	-0.2474	0	0.138	0.000169		131	Aff1	2.04E-08	-0.36493	0.298	0.455	0.000422
132	Adam8	8.47E-09	-0.46694	0.579	0.801	0.000175		132	Gm4951	2.33E-08	-0.3524	0.071	0.229	0.000482
133	Tspo	8.53E-09	-0.28265	0.979	0.987	0.000176		133	Ctss	2.45E-08	-0.36062	0.949	0.997	0.000506
134	Tbc1d22a	8.59E-09	0.186284	0.262	0.198	0.000177		134	Slpi	2.47E-08	-0.99025	0.328	0.573	0.00051
135	Slc16a3	8.68E-09	-0.44971	0.531	0.739	0.000179		135	Rps6	2.47E-08	0.317194	0.889	0.855	0.000511
136	Uri1	8.89E-09	0.137253	0.179	0.156	0.000183		136	Zdhhc14	2.50E-08	0.164524	0.157	0.046	0.000516
137	Ubb	9.56E-09	-0.36582	0.938	0.961	0.000197		137	Serpinb6a	2.72E-08	0.480587	0.364	0.216	0.000562
138	Rnf128	1.03E-08	-0.36361	0.09	0.302	0.000213		138	Trf	3.02E-08	0.620052	0.399	0.224	0.000624
139	Eif3e	1.31E-08	0.230467	0.662	0.649	0.00027		139	Laptm5	3.28E-08	0.321098	0.904	0.903	0.000677
140	Coro2a	1.36E-08	-0.25649	0.152	0.398	0.00028		140	Ly6c1	3.38E-08	-0.39324	0.066	0.244	0.000698
141	Pdia4	1.56E-08	-0.3206	0.51	0.588	0.000322		141	Epb41l1	3.92E-08	0.215337	0.197	0.071	0.000809
142	Ctsc	1.60E-08	0.281436	0.972	0.953	0.000331		142	Ifi27l2a	4.12E-08	-0.47059	0.813	0.896	0.000851
143	Arl4a	1.64E-08	0.127691	0.248	0.253	0.000339		143	Ccdc115	4.43E-08	0.233905	0.293	0.232	0.000914
144	F10	1.67E-08	-0.49429	0.241	0.503	0.000345		144	Tnfrsf1b	5.11E-08	-0.43039	0.631	0.753	0.001056
145	Rps28	1.70E-08	0.246963	1	0.984	0.000352		145	Rps23	5.31E-08	0.27708	0.949	0.941	0.001097
146	Cd68	1.81E-08	-0.3849	0.862	0.933	0.000374		146	Met	5.41E-08	-0.28756	0.172	0.389	0.001116
147	B2m	1.89E-08	-0.21964	1	1	0.000391		147	Cd38	5.42E-08	-0.42139	0.162	0.361	0.001119
148	Esd	2.01E-08	-0.39661	0.669	0.85	0.000416		148	mt-Cytb	5.53E-08	0.417152	0.97	0.934	0.001141
149	C1qa	2.09E-08	0.592959	0.745	0.621	0.000433		149	Fn1	6.01E-08	0.762499	0.657	0.514	0.001241
150	Cadm1	2.14E-08	0.36125	0.324	0.118	0.000442		150	Apoe	6.09E-08	0.43547	0.803	0.677	0.001258

TABLE S2 (CONT.)

	Macrophage Cluster 3					
		p_val	avg_logFC	pct.1	pct.2	p_val_adj
151	Sp110	2.50E-08	0.21259	0.579	0.632	0.000516
152	Pfdn5	2.58E-08	0.257302	0.91	0.897	0.000532
153	Pnn	2.61E-08	0.125198	0.262	0.281	0.00054
154	Tppp3	2.75E-08	-0.41012	0.2	0.446	0.000567
155	Samm50	2.81E-08	0.219159	0.455	0.35	0.00058
156	Usp48	2.85E-08	0.171512	0.276	0.232	0.000588
157	Mdm2	3.02E-08	-0.43529	0.552	0.747	0.000624
158	Ccl2	3.19E-08	-0.62072	0.834	0.929	0.000658
159	Ppp1r21	3.59E-08	0.206143	0.359	0.289	0.000741
160	Rpl8	3.64E-08	0.197088	0.993	0.98	0.000752
161	Cd300lf	3.71E-08	-0.43749	0.49	0.688	0.000766
162	Ubc	4.04E-08	-0.37066	0.876	0.951	0.000833
163	Cpsf4	4.08E-08	0.109321	0.234	0.255	0.000842
164	Anxa4	4.24E-08	-0.31439	0.759	0.889	0.000875
165	Tgfbr2	4.54E-08	0.225531	0.469	0.433	0.000937
166	Slc40a1	4.55E-08	0.183359	0.069	0.119	0.000939
167	Anapc1	4.59E-08	0.160148	0.324	0.284	0.000947
168	Slpi	4.95E-08	-0.90625	0.2	0.398	0.001021
169	Ankrd11	4.97E-08	0.306525	0.662	0.539	0.001026
170	Gpr132	5.35E-08	0.317914	0.448	0.387	0.001104
171	Tnf	5.65E-08	-0.49004	0.469	0.701	0.001166
172	C6	5.99E-08	0.21951	0.103	0.009	0.001237
173	Arfgap3	6.29E-08	0.109269	0.207	0.217	0.001299
174	Tmem59	6.47E-08	0.182429	0.628	0.667	0.001336
175	Hs6st1	6.51E-08	0.170633	0.145	0.106	0.001345
176	Ccar1	6.87E-08	0.163016	0.359	0.346	0.001419
177	Tuba1c	7.11E-08	-0.27446	0.621	0.836	0.001468
178	Inhba	7.30E-08	-0.77315	0.11	0.323	0.001508
179	Glipr2	7.39E-08	-0.333	0.324	0.58	0.001526
180	Synrg	7.50E-08	0.156323	0.241	0.185	0.001548
181	Rhob	7.83E-08	-0.24056	0.517	0.762	0.001616
182	Utp11	9.69E-08	0.126404	0.324	0.347	0.002002
183	Sowahc	9.82E-08	0.301563	0.428	0.448	0.002027
184	A930007I1	1.08E-07	-0.25333	0.145	0.366	0.002238
185	Irf7	1.21E-07	-0.20512	0.793	0.929	0.002506
186	Crip1	1.24E-07	-0.6451	0.924	0.954	0.00256
187	Slc9a9	1.34E-07	0.235153	0.29	0.165	0.002759
188	Rps14	1.34E-07	0.223498	0.986	0.982	0.002762
189	Mbnl1	1.41E-07	0.306969	0.814	0.699	0.002918
190	Rps21	1.42E-07	0.224764	0.993	0.978	0.002924
191	Cttnbp2nl	1.54E-07	0.221724	0.49	0.422	0.003187
192	Fuca1	1.60E-07	0.199508	0.538	0.516	0.003308
193	Zfand5	1.74E-07	-0.32951	0.745	0.895	0.003588
194	Sectm1a	1.76E-07	0.261268	0.29	0.211	0.003642
195	Rps13	1.83E-07	0.257596	0.979	0.962	0.003769
196	Utp14b	1.87E-07	0.124301	0.117	0.048	0.003867
197	Clptm1	1.91E-07	0.133777	0.434	0.456	0.003939
198	Socs1	1.94E-07	-0.29097	0.393	0.646	0.004006
199	Akap8	1.99E-07	0.189974	0.29	0.175	0.004112
200	Cxcl2	2.03E-07	-0.61621	0.786	0.9	0.004202

	Macrophage Cluster 5					
		p_val	avg_logFC	pct.1	pct.2	p_val_adj
151	Plpp3	6.41E-08	-0.49026	0.187	0.407	0.001325
152	Rpl18	6.43E-08	0.296077	0.97	0.954	0.001328
153	Rps9	8.47E-08	0.245806	1	0.99	0.001749
154	Comt	9.80E-08	0.205825	0.217	0.127	0.002023
155	Pde3b	1.04E-07	0.293506	0.258	0.115	0.002154
156	Tpt1	1.35E-07	0.144965	1	0.995	0.002779
157	Plau	1.38E-07	0.601371	0.586	0.425	0.002855
158	Rps21	1.42E-07	0.207241	0.99	0.962	0.002925
159	mt-Nd4	1.55E-07	0.365023	0.914	0.919	0.003209
160	Iigp1	1.80E-07	-0.71447	0.152	0.361	0.003717
161	S100a8	2.03E-07	-0.76386	0.273	0.483	0.004193
162	Rps18	2.04E-07	0.342766	0.823	0.735	0.004203
163	Ms4a4c	2.09E-07	-0.38299	0.263	0.504	0.004309
164	Ybx1	2.21E-07	0.24816	0.929	0.898	0.00457
165	Tap1	2.35E-07	-0.3726	0.515	0.682	0.004852
166	Rpl6	2.61E-07	0.266011	0.949	0.949	0.005395
167	Atp5g2	2.70E-07	0.29313	0.843	0.786	0.005577
168	Kmt5a	2.87E-07	-0.35284	0.328	0.55	0.005926
169	Rgs10	3.15E-07	0.30282	0.495	0.354	0.006495
170	Npm1	3.24E-07	0.287231	0.874	0.832	0.006692
171	Ptma	3.30E-07	0.296421	0.975	0.947	0.006806
172	Plin2	3.84E-07	-0.3708	0.914	0.977	0.007932
173	1110038F1	4.11E-07	-0.18827	0.167	0.254	0.008494
174	H2-T22	4.70E-07	-0.3651	0.495	0.616	0.009698
175	Ell2	4.72E-07	-0.36987	0.369	0.608	0.009752
176	Cfp	4.92E-07	0.381575	0.727	0.55	0.010162
177	Rpl39	5.00E-07	0.234487	0.965	0.949	0.01033
178	Rpl35	5.31E-07	0.2344	0.864	0.733	0.010964
179	Rps17	5.70E-07	0.269756	0.869	0.819	0.011776
180	Il7r	5.94E-07	-0.40664	0.717	0.865	0.012269
181	Rapgef2	6.24E-07	-0.47042	0.439	0.593	0.012882
182	Irf1	6.49E-07	-0.49164	0.581	0.723	0.01341
183	Saa3	6.68E-07	-1.62843	0.056	0.209	0.013792
184	Fam168a	6.80E-07	0.137339	0.258	0.267	0.014049
185	Daglb	6.87E-07	0.18372	0.222	0.135	0.014178
186	Rpl10	7.00E-07	0.263651	0.914	0.916	0.014453
187	Lgals3	7.32E-07	-0.12765	0.985	0.992	0.015121
188	Cd81	7.56E-07	0.356567	0.308	0.122	0.015619
189	Psmb8	8.03E-07	-0.34445	0.692	0.802	0.016589
190	Cyb5b	9.13E-07	0.135842	0.177	0.135	0.018862
191	Nr4a1	9.21E-07	0.529843	0.556	0.387	0.019021
192	Xaf1	1.00E-06	-0.28425	0.177	0.392	0.020734
193	Hspa8	1.04E-06	0.380006	0.965	0.936	0.02156
194	Mef2c	1.07E-06	0.293097	0.268	0.092	0.022011
195	Fos	1.15E-06	0.356499	0.874	0.919	0.023838
196	Cat	1.18E-06	0.28181	0.338	0.176	0.024277
197	Cct2	1.35E-06	0.243684	0.48	0.354	0.027889
198	Ctsh	1.36E-06	-0.34692	0.712	0.847	0.02815
199	Ski	1.45E-06	0.272548	0.424	0.336	0.029934
200	Scaf8	1.45E-06	0.177446	0.182	0.158	0.030003

TABLE S2 (CONT.)

	Macrophage Cluster 3					
		p_val	avg_logFC	pct.1	pct.2	p_val_adj
201	Stx11	2.13E-07	-0.21156	0.255	0.497	0.004391
202	Atp6v1a	2.15E-07	-0.2689	0.724	0.897	0.004438
203	Clock	2.15E-07	0.145801	0.269	0.245	0.00444
204	Slc25a1	2.18E-07	0.106117	0.262	0.318	0.004503
205	Ifitm6	2.31E-07	-0.32974	0.103	0.309	0.00477
206	Pcmtd1	2.34E-07	0.232332	0.483	0.395	0.004838
207	Laptm5	2.35E-07	0.228208	0.979	0.98	0.004851
208	Mark2	2.36E-07	0.102561	0.359	0.395	0.004867
209	S100a8	2.56E-07	-0.58237	0.221	0.465	0.005288
210	Eef2	2.59E-07	0.274754	0.924	0.906	0.00535
211	Ifi30	2.62E-07	0.317628	0.924	0.883	0.005415
212	Tarm1	2.77E-07	-0.28328	0.193	0.396	0.005722
213	Creg1	2.97E-07	-0.41984	0.821	0.904	0.006126
214	Tifab	3.05E-07	0.231532	0.455	0.371	0.006301
215	2410006H	3.16E-07	0.267389	0.455	0.37	0.006524
216	Tcf4	3.18E-07	0.30895	0.662	0.558	0.006573
217	Acod1	3.19E-07	-0.54614	0.593	0.772	0.006577
218	Nos2	3.22E-07	-0.70414	0.166	0.349	0.006645
219	Rps18	3.25E-07	0.245493	0.848	0.825	0.006713
220	mt-Nd2	3.38E-07	0.336176	0.952	0.938	0.006987
221	Rpl9	3.40E-07	0.244205	0.993	0.963	0.007027
222	Fbxl5	3.51E-07	-0.45623	0.538	0.705	0.007249
223	Plekhm1	3.54E-07	-0.26274	0.193	0.427	0.007311
224	Trappc3	3.71E-07	0.139541	0.393	0.403	0.007666
225	H2-DMb2	3.80E-07	0.227344	0.331	0.191	0.007843
226	Igf2r	3.97E-07	-0.14298	0.138	0.32	0.008198
227	Atp6v1c1	4.13E-07	-0.27817	0.552	0.764	0.008519
228	Eif3k	4.36E-07	0.24139	0.883	0.84	0.008997
229	mt-Co2	4.43E-07	0.217088	0.979	0.993	0.009141
230	Top2b	4.65E-07	0.20273	0.386	0.318	0.009612
231	Itgb7	4.78E-07	-0.37717	0.31	0.535	0.009877
232	Tagln2	4.81E-07	-0.40653	0.772	0.871	0.009929
233	Lilrb4a	4.90E-07	-0.32507	0.834	0.937	0.010114
234	Evi2a	5.42E-07	0.206481	0.641	0.641	0.011199
235	Stag2	5.83E-07	0.191359	0.428	0.403	0.012045
236	Rpl22l1	5.98E-07	0.266161	0.752	0.742	0.012356
237	Mtmr6	6.04E-07	0.126478	0.331	0.354	0.012464
238	Il6ra	6.22E-07	0.286241	0.6	0.454	0.012839
239	Txnrd1	6.53E-07	-0.28853	0.455	0.674	0.013474
240	Rpsa	6.71E-07	0.27361	0.993	0.974	0.013848
241	Rpl11	6.73E-07	0.251423	0.966	0.965	0.013889
242	Irf1	6.77E-07	-0.29562	0.8	0.932	0.013975
243	Rnh1	6.84E-07	-0.30913	0.697	0.861	0.014122
244	Ppp3ca	7.31E-07	0.291134	0.655	0.562	0.015094
245	Klf6	7.45E-07	-0.3064	0.945	0.986	0.015373
246	Lhfpl2	7.46E-07	-0.38135	0.207	0.399	0.015398
247	Hltf	7.46E-07	0.125651	0.2	0.152	0.015399
248	Ifi203	7.74E-07	0.331131	0.703	0.73	0.015984
249	Cysltr1	7.81E-07	0.254476	0.51	0.395	0.016129
250	Bst1	7.93E-07	-0.28952	0.579	0.767	0.01637

	Macrophage Cluster 5					
		p_val	avg_logFC	pct.1	pct.2	p_val_adj
201	C1qa	1.54E-06	0.736931	0.48	0.308	0.031772
202	Plec	1.56E-06	0.16778	0.495	0.545	0.032295
203	Rpl30	1.61E-06	0.227803	0.965	0.959	0.033217
204	Lamp1	1.72E-06	0.248966	0.97	0.969	0.035508
205	Ifitm3	1.77E-06	-0.31641	0.929	0.977	0.036566
206	Ass1	1.90E-06	-0.30642	0.131	0.328	0.039252
207	Kdm1a	1.95E-06	0.271397	0.389	0.27	0.040255
208	Fmnl2	2.11E-06	-0.29051	0.374	0.509	0.043629
209	Stx11	2.14E-06	-0.31171	0.293	0.433	0.044137
210	Dtx3l	2.31E-06	-0.32028	0.227	0.435	0.047715
211	Rpl35a	2.60E-06	0.179422	0.975	0.98	0.053763
212	Rpl4	2.63E-06	0.2647	0.722	0.669	0.054347
213	C1qc	2.99E-06	0.640487	0.465	0.316	0.061662
214	Atp2c1	3.11E-06	0.124977	0.207	0.198	0.064222
215	Zranb3	3.45E-06	0.130833	0.141	0.069	0.071304
216	Fkbp1a	3.47E-06	0.266805	0.788	0.753	0.071679
217	Ly6i	3.49E-06	-0.24818	0.025	0.155	0.072056
218	Gbp6	3.50E-06	-0.33379	0.116	0.244	0.072181
219	Ubb	3.58E-06	-0.20862	0.955	0.975	0.073856
220	Ccl5	3.66E-06	-1.10073	0.106	0.282	0.075659
221	Srgn	3.94E-06	-0.38888	0.99	0.972	0.08144
222	Acod1	4.10E-06	-0.25192	0.48	0.7	0.084697
223	Nrp1	4.16E-06	0.275088	0.237	0.109	0.085932
224	Spg7	4.30E-06	0.147077	0.157	0.135	0.088704
225	Tcp11l2	4.43E-06	0.278662	0.293	0.176	0.091419
226	Hcar2	4.65E-06	-0.39147	0.308	0.527	0.095931
227	Ptafr	4.89E-06	-0.38782	0.49	0.687	0.100976
228	Trim47	4.97E-06	0.150737	0.131	0.061	0.102607
229	Ncf1	5.20E-06	0.360422	0.49	0.364	0.107421
230	Ssr3	5.48E-06	-0.3254	0.657	0.768	0.113107
231	LTO1	5.81E-06	0.103705	0.131	0.104	0.119986
232	Psme1	5.92E-06	-0.30207	0.677	0.809	0.122242
233	Pitpnc1	6.47E-06	0.346048	0.394	0.234	0.133535
234	mt-Nd2	6.59E-06	0.362287	0.793	0.746	0.136006
235	Cd52	6.82E-06	-0.38167	0.687	0.824	0.140851
236	Itm2b	6.89E-06	0.248282	0.924	0.936	0.142278
237	Ntpcr	6.99E-06	0.108637	0.187	0.122	0.144332
238	Mapk3	7.00E-06	0.18844	0.54	0.532	0.144497
239	Tspo	7.00E-06	-0.2652	0.914	0.939	0.144543
240	Txn1	7.41E-06	-0.28481	0.899	0.962	0.153087
241	Rpl37	8.17E-06	0.198732	0.97	0.952	0.168679
242	Rp9	8.44E-06	0.256958	0.52	0.438	0.174204
243	Stxbp5	8.60E-06	0.20242	0.217	0.137	0.177491
244	Ifit1	8.77E-06	-0.41726	0.096	0.267	0.18101
245	Ak2	8.78E-06	-0.28717	0.374	0.527	0.181298
246	Slfn2	8.98E-06	-0.25404	0.788	0.913	0.185517
247	Gng2	8.99E-06	0.281464	0.662	0.601	0.185656
248	Akap13	9.28E-06	-0.43739	0.586	0.733	0.191718
249	Rcbtb2	9.41E-06	0.297642	0.419	0.244	0.194243
250	Clk1	9.74E-06	0.237196	0.636	0.573	0.201205

Table S2: Differentially expressed genes between exoASO-STAT6 and control exoASO groups in macrophage cluster 3 and macrophage cluster 5

TABLE S3

Naïve C57Bl/6 Cytokines	exoASO-STAT6 (4h)			exoASO Scramble (4h)			exoASO-STAT6 (4 days)			exoASO Scramble (4 days)		
MCP-1	202.5	±	17.2	406.1	±	84.1	8.6	±	0.2	10.0	±	0.6
IL1B	24.7	±	8.5	17.3	±	5.7	19.0	±	3.1	5.6	±	0.6
IL23	37.9	±	25.6	23.2	±	5.2	11.4	±	3.7	12.4	±	5.5
IFN-γ	175.9	±	45.4	151.1	±	45.5	2.1	±	1.1	0.9	±	0.2
IL6	9.7	±	1.1	13.5	±	2.4	1.1	±	0.2	0.7	±	0
IL-17A	1.2	±	0.2	2.0	±	0.5	1.6	±	0.5	1.0	±	0.2

Table S3: Cytokine evaluation in serum (in pg/ml) of the listed cytokines, using a multiplex flow cytometry analysis, from naïve C57Bl/6 mice injected with a single dose of exoASO-Scramble (12ug) or exoASO-STAT6-2039 (PTGFRN++) (12ug), data was analyzed at 4 hours and 4 days post injection.

TABLE S4

(HEPA1-6) Cytokines	exoASO-STAT6			exoASO Scramble			Sham (no tumor)		
IL-23	11.4	±	0.0	11.4	±	0.0	10.0	±	2.8
IL-1a	6.7	±	2.5	7.8	±	8.4	11.5	±	19.3
IFN-γ	172.5	±	127.8	167.8	±	69.8	12.4	±	8.8
TNF-α	11.1	±	9.6	20.5	±	7.2	2.6	±	1.8
MCP-1	56.2	±	41.5	195.4	±	85.2	35.4	±	26.1
IL-12p70	2.1	±	0.0	2.1	±	0.0	1.8	±	0.5
IL-1β	4.1	±	4.5	3.1	±	2.6	1.5	±	0.4
IL-10	2.4	±	0.5	3.6	±	3.8	2.0	±	0.6
IL-6	4.8	±	1.6	12.6	±	20.8	3.7	±	1.1
IL-27	19.9	±	0.0	32.9	±	25.9	17.4	±	5.0
IL-17A	54.7	±	137.9	5.0	±	3.4	9.1	±	11.9
IFN-β	138.7	±	318.6	31.7	±	16.1	22.8	±	6.5
GM-CSF	1.2	±	0.0	1.2	±	0.0	1.0	±	0.3

Table S4: Cytokine evaluation in serum (in pg/ml) of the listed cytokines, using a multiplex flow cytometry analysis, from Hepa1-6 mice from (**Fig 5A-J**). Sham mice underwent the same inoculation procedures as experimental mice and were used as no-tumor controls

TABLE S5

No.	Gene
1	CD163
2	IL4R
3	TGFb1
4	MRC1
5	AIF1
6	CD40
7	CD276
8	MIF
9	CCL26
10	COL1A2
11	ALOX15
12	SELE
13	CEBPB
14	MYC
15	CD44
16	SIGLEC 1
17	CD274
18	CCL13
19	CCL5
20	CXCL9
21	CTSS
22	CSF1R
23	C1QA
24	C1QB
25	C1QC
26	CD68
27	FN1
28	CD14
29	CCL2
30	CCR3
31	IL1B
32	CD1A
33	EDN1
34	IL15RA
35	IRF5
36	IL12B
37	CD86
38	CCR7
39	PTGS1
40	CCL17

Table S5: Macrophage STAT6 gene signature
